# *In vitro* effects of European and Latin-American medicinal plants in CYP3A4 gene expression, glutathione levels, and P-glycoprotein activity

**DOI:** 10.3389/fphar.2022.826395

**Published:** 2022-10-05

**Authors:** Andre Luis Dias Araujo Mazzari, Mariella Guimarães Lacerda, Flora Aparecida Milton, João Augusto Mulin Montechiari Machado, Simone Batista Pires Sinoti, Anne-Soulene Toullec, Patricia Marquez Rodrigues, Francisco de Assis Rocha Neves, Luiz Alberto Simeoni, Dâmaris Silveira, Jose Maria Prieto

**Affiliations:** ^1^ School of Pharmacy, University College London, London, United Kingdom; ^2^ Faculdade de Ciências da Saúde, Universidade de Brasília, Brasília, Brazil; ^3^ Instituto de Saúde de Nova Friburgo, Universidade Federal Fluminense, Niterói, Brazil; ^4^ School of Pharmacy and Biomolecular Sciences, Liverpool John Moores University, Liverpool, United Kingdom

**Keywords:** herbal medicine, Brazil, Europe, pharmacokinetics, preclinical drug evaluation, drug metabolism, toxicology

## Abstract

Many medicinal plants species from European -such as *Artemisia absinthium, Equisetum arvense, Lamium album, Malva sylvestris, Morus nigra, Passiflora incarnata, Frangula purshiana, and Salix alba*- as well as Latin American traditions -such as *Libidibia ferrea, Bidens pilosa, Casearia sylvestris, Costus spicatus, Monteverdia ilicifolia, Persea americana, Schinus terebinthifolia, Solidago chilensis*, *Syzygium cumini, Handroanthus impetiginosus,* and *Vernonanthura phosphorica-* are shortlisted by the Brazilian National Health System for future clinical use. However, they lack many data on their action upon some key ADME targets. In this study, we assess non-toxic concentrations (up to100 μg/ml) of their infusions for *in vitro* ability to modulate CYP3A4 mRNA gene expression and intracellular glutathione levels in HepG2 cells, as well as P-glycoprotein (P-gp) activity in vincristine-resistant Caco-2 cells (Caco-2 VCR). We further investigated the activation of human pregnane X receptor (hPXR) in transiently co-transfected HeLa cells and the inhibition of Gamma-glutamyl transferase (GGT) in HepG2 cells. Our results demonstrate *L. ferrea, C. sylvestris* *, M. ilicifolia, P. americana, S. terebinthifolia, S. cumini, V. phosphorica, E. arvense, P. incarnata, F. purshiana,* and *S. alba* can significantly increase CYP3A4 mRNA gene expression in HepG2 cells. Only *F. purshiana* shown to do so likely via hPXR activation. P-gp activity was affected by *L. ferrea, F. purshiana, S. terebinthifolia,* and *S. cumini*. Total intracellular glutathione levels were significantly depleted by exposure to all extracts except *S. alba* and *S. cumini* This was accompanied by a lower GGT activity in the case of *C. spicatus, P. americana, S. alba,* and *S. terebinthifolia*, whilst *L. ferrea*, *P. incarnata* and *F. purshiana* increased it. Surprisingly, *S. cumini* aqueous extract drastically decreased GGT activity (−48%, *p* < 0.01). In conclusion, this preclinical study shows that the administration of some of these herbal medicines causes *in vitro* disturbances to key drug metabolism mechanisms. We recommend active pharmacovigilance for *Libidibia ferrea* (Mart.) L. P. Queiroz, *Frangula purshiana* Cooper, *Schinus terebinthifolia* Raddi, and *Salix alba* L. which were able to alter all targets in our preclinical study.

## 1 Introduction

The profile of pharmaceutical drugs at both the absorption and metabolic levels is crucial to determine whether they will interact with therapeutic efficiency (Chen et al., 2012). However, studies on metabolic and transporter targets using medicines based on herbal drug extracts (or “herbal preparations”) are challenging to carry out because of their chemical complexity (Li et al., 2019). In fact, there is scarce information about the pharmacokinetics (PK) effects they could cause on phase 1 and phase 2 metabolism and P-glycoprotein (P-gp). Such data are particularly relevant to predict and avoid interactions with conventional drugs, the so-called herb-drug interactions (HDI).

Latin American countries possess an enormous part of the world’s biodiversity, with numerous plant species containing medicinal properties (Mazzari et al., 2016). Brazil can be highlighted with around 55,000 species (Savi et al., 2019) and the public policies for introducing Herbal Medicines and other Complementary Practices in the public health system (SUS—Unified Health System), such as National Policy for Complementary and Integrative Practices (PNPIC) and National Policy for Medicinal Plants and Herbal Medicines (PNPMF), both launched in 2006 (BRASIL, 2006a; BRASIL, 2006b). The Brazilian Health Surveillance System, coordinated by Brazilian Health Regulatory Agency (ANVISA), is responsible for assessing the herbal medicine industry in the country, meaning all issues related to marketing, dispensing, handling and distribution of raw materials of plant origin, as well as the registration and supervision of herbal medicine production.

In 2009, The Ministry of Health published the List of Species of Interest for The Unified Health System (RENISUS) (BRASIL, 2009) to stimulate researchers and the pharmaceutical industry to develop herbal medicines. And a few years later, ANVISA, through the Brazilian Pharmacopeia, launched the National Formulary of Herbal Remedies (BRASIL, 2021), and the “Memento of Herbal Medicines” (BRASIL, 2016), to support health workers and pharmaceutical industry on the prescription of herbal medicines in SUS. These two official documents bring information about herbal remedies and herbal drugs from several Latin American species; some of them are included in the RENISUS (BRASIL, 2009), and allowed to be prescribed to Brazilian patients (Table 1): *Libidibia ferrea* (Mart.) L. P. Queiroz. (accepted name of *Apuleia ferrea* (Mart.) Bail), *Bidens pilosa* L., *Casearia sylvestris* Sw., *Costus spicatus* (Jacq.) Sw., *Monteverdia ilicifolia* (Mart. ex Reissek) Biral (accepted name of *Maytenus ilicifolia* Mart. Ex Reissek), *Persea americana* Mill., *Schinus terebinthifolia* Raddi, *Solidago chilensis* Meyen (accepted name of *Solidago microglossa* DC.), *Syzygium cumini* (L.) Skeels [accepted name of *Syzygium jambolanum* (Lam.) DC.], *Handroanthus impetiginosus* (Mart. Ex DC.) Mattos (accepted name of *Tabebuia avellanedae* Lorentz ex Griseb.), and *Vernonanthura phosphorica* (Vell.) H. Rob. (accepted name of *Vernonia polyanthes* Less.) (BRASIL, 2016, BRASIL, 2021).

**TABLE 1:** Summary of the traditional indications and chemical composition of the selected Latin American herbal medicines.

*Plant species* (family) herbal drug	Traditional indication	Registered medicinal products/Indication	Phytochemistry
*Apuleia ferrea* (Mart.) Baill (Fabaceae)	Wound healing, antiseptic BRASIL (2021)	—	Gallic acid^a^ ^,^ ^b^, catechin, epicatechin and ellagic acid Vasconcelos et al. (2011); valoneic acid; filactone; 3-O-galloyl quinic acid; ethyl gallate; methyl ellagic acid-pentoside Wyrepkowski et al. (2014); pauferrol A Nozaki et al. (2007); pauferrol B; pauferrol C Ohira et al. (2013); polysaccharide^a^ Pereira et al. (2012); eriodictyol-O-hexoside; naringenin-O-hexoside; campesterol Ferreira et al. (2019)
Fruits			
Accepted name: *Libidibia ferrea* (Mart.) L. P. Queiroz			
*Bidens pilosa* L. (Asteraceae)	Jaundice BRASIL (2021)	—	Rutin; hyperoside; 4,5-O-dicaffeoylquinic acid Cortés-Rojas et al. (2013)
Aerial parts			
*Casearia sylvestris* Sw. (Salicaceae)	Dispepsia BRASIL (2021)	—	Essential oil (major components: caryophyllene, acoradiene, bicyclogermacrene)^a^ Esteves et al. (2005)
Leaves			
*Costus spicatus* (Jacq.) Sw^c^.	Diuretic, emenagogue and diaforetic Lorenzi and Matos (2002)	—	Quercetin; tamarixetin glycosides; furostanol glycosides Silva et al. (1999); Silva et al. (2000)
(Costaceae)			
Leaves			
*Maitenus ilicifolia* Mart. ex Reissek (Celastraceae)	Dispepsia, anti-acid BRASIL, (2016), BRASIL (2021); gastroprotective BRASIL (2016)	Capsules, tincture/anti-ulcer, anti-acid	Friedelanol; Queiroga et al. (2000); maytenin, pristimerin, celastrol Périco et al. (2018); pirogalol, epicatechin^b^ BRASIL (2019)
Leaves			
Accepted name *Monteverdia ilicifolia* (Mart. ex Reissek Biral)			
*Persea americana* Mill. (Lauraceae)	Diuretic BRASIL (2021)	Capsules/osteoarthritis pain	Apigenin^b^, rutin^b^ BRASIL (2019)
Leaves		Tincture/anti-inflammatory	
*Schinus terebinthifolia* Raddi (Anacardiaceae)	Anti-inflammatory; wound healing (gynecology) BRASIL, (2011)	Tablets/anti-ulcer; gel/gynaecological, anti-microbial	Gallic acid^b^, pirogalol^b^, catechin2 BRASIL (2019)
Bark			
*Solidago microglossa* DC. (Asteraceae)	Anti-inflammatory; wound healing^c^ Lorenzi and Matos (2002)	—	Solidagenone Valverde et al. (2009); quercitrin Vechia et al. (2016)
Aerial parts			
Accepted name: *Solidago chilensis* Meyen			
*Syzygium jambolanum* (Lam.) DC.	Hypoglycemiant^c^ Lorenzi and Matos (2002)	—	Jambosine, ellagic acid and gallic acid Chagas et al. (2014)
(Myrtaceae)			
Fruits and seeds			
Accepted name: *Syzygium cumini* (L.) Skeels			
*Tabebuia avellanedae* Lorentz ex Griseb (Bignoniaceae)	Anti-inflammatory (BRASIL, (2018)	—	Lapachol^a^ and β-lapachone^a^ Cragg et al. (2010)
Bark			
Accepted name: *Handroanthus impetiginosus* (Mart. Ex DC.) Mattos			
*Vernonia polyanthes* Less. (Asteraceae)	Expectorant BRASIL (2011)	—	Quercetin^b^, luteolin^b^, Kaempferol^b^ BRASIL (2011)
Leaves			
Accepted name: *Vernonanthura phosphorica* (Vell.) H.Rob			

a Pharmacological active principle.

b Pharmacopoeial phytomarker.

c Documented traditional Brazilian use at the time of its inclusion in RENISUS.

This list also included many herbal medicines from the European tradition such as *Artemisia absinthium* L., *Equisetum arvense* L., *Lamium album* L., *Malva silvestris* L., *Morus nigra* L., *Passiflora incarnata* L., *Frangula purshiana* Cooper (accepted name of *Rhamnus purshiana* L), and *Salix alba* L. (BRASIL, 2009), most of them still officially endorsed by monographs of the European Medicines Agency (EMA) as eligible for the registration of Traditional Herbal Medicinal Products in any country member of the Union under the Directive 2001/83/EC (Table 2).

**TABLE 2:** Summary of the traditional indications and chemical composition of the selected European herbal medicines.

Plant species (family) part of the plant	Traditional indication	Registered medicinal products/Indication	Phytochemistry
*Artemisia absinthium* L. (Asteraceae)	Temporary loss of appetite; mild dyspeptic/gastrointestinal disorders EMA, (2020a)	—	essential oil^a^ (a-pinene, myrcene, camphor, terpinene-4-ol, thujone2) Aati et al., (2020), artemisinin^b^, absintins, quercetin derivatives Batiha et al., (2020)
Leaves or leafy flowering tops			
*Equisetum arvense* L. (Equisetaceae)	Diuretic; wound healing BRASIL, (2016); EMA, (2016); BRASIL, (2021)	Capsules/diuretic	kaempferol, quercetin and luteolin derivatives, caffeic acid derivatives, campsterol, isofucosterol, silicic acid Al-Snafi (2017); Boeing et al. (2021)
Aerial parts			
*Lamium album* L. (Lamiaceae)	Astringent and haemostatic^c^ Williamson and Wren, (2003)	—	Isoscutellarein derivatives, quercetin derivatives, verbascoside, isoverbascoside, lamalbosid, butasterone, inokosterone, polypodine B, pterosterone Shah et al. (2019)
Leaves			
*Malva silvestris* L. (Malvaceae)	Oral or pharyngeal irritation associated dry cough; mild gastrointestinal discomfort EMA (2018); BRASIL (2021)	—	Malvone A, delphinidine, quercetin, myricetin, apigenin, genistein, kaempferol Mousavi et al. (2021)
Leaves			
*Morus nigra* L. (Moraceae)	Laxative, diuretic, hypotensive, expectorant and reduction of blood sugar levels^c^ Williamson and Wren (2003)	—	Flavonoids Lim and Choi (2019)
Leaves			
*Passiflora incarnata* L. (Passifloraceae)	Mild mental stress; insomnia EMA, (2014); BRASIL (2021); anxyolitic; sedative Brasil (2016)	Tablets/anxyolitic	Vitexin, isovitexin, orientin, isoorienti, kaempferol, schaftoside, isoschaftoside, swertisin, chrysin, apigenin, luteolin, Harman, harmol, harmine, harmalol, harmaline Khan and Nabavi (2018)
Aerial parts			
*Rhamnus purshiana* L. (Rhamnaceae)	Occasional constipation Brasil (2016); EMA, (2020b)	Capsules/laxative	Cascarosides A-F Demarque et al., (2017)
Accepted name: *Frangula purshiana* Cooper			
Bark			
*Salix alba* L. (Salicaceae)	Analgesic, anti-inflammatory, febrifuge and tonic^c^ (Brasil (2011); Relief of minor articular pain, fever associated with common cold, and headache EMA, (2017)	Capsules, elixir/analgesic, anti-inflammatory	Salicin EMA (2017)
Bark			

a Pharmacological active principle.

b Pharmacopoeial phytomarker.

c This herbal medicinal drug is included in the RENISUS, list of clinically relevant medicinal herbal medicines, but it is not listed in current official pharmacopoeias, phytotherapy monographs (Memento) or EMA, monograph.

Their medicinal uses and chemical composition are scientifically backed up (Tables 1, 2) but there is scarce information on their influence on drug metabolism. Thus, a potential risk for herbal-drug interaction for some of these herbal drugs exist as reported in our previous works (Mazzari and Prieto, 2014; Mazzari et al., 2016). Therefore, this study aims to contribute new pre-clinical data on this matter by investigating the *in vitro* effects of these European and Latin-American medicinal plants on CYP3A4 mRNA gene expression, activation of the human pregnane X receptor (hPXR), intracellular glutathione levels, Gamma-glutamyl transferase (GGT), and P-glycoprotein (P-gp) activity.

## 2 Materials and methods

### 2.1 Chemicals and reagents

#### 2.1.1 Cell culture

HepG2 and Caco-2 cell lines were from Sigma Aldrich. HeLa cells were donated by Dr. Paul Webb from the Houston Methodist Institute for Technology at passage 20. All cell culture reagents were from Gibco^®^ Invitrogen unless otherwise stated. Vincristine 2 mg/ml was purchased from Hospira Ltd.

#### 2.1.2 HPLC analysis

Water and methanol all ChromasolvPlus for HPLC provided by Sigma Aldrich. Acetic acid (glacial) analytical reagent grade provided by Fisher Scientific. Formic Acid 98% provided by Rectapur^®^ VWR.

Caffeic acid was from Kotch-light Laboratories LTD. Rutin hydrate, quercetin dehydrate, and gallic acid all from Sigma Aldrich. Diphenylborinic acid 2-aminoethyl ester (98%) were provided by ACROS organics. Luteolin (HPLC grade) was from Extrasynthése. Chlorogenic acid provided by Cayman Chemical Co. Polyethylene glycol 4000 grade was from Fisher Scientific.

#### 2.1.3 Cell viability assays

Sulphorhodamine B (SRB), Neutral Red (NR), (3-[4,5-dimethylthiazol-2-yl]-2,5 diphenyl tetrazolium bromide) (MTT), trichloroacetic acid (TCA), glacial acetic acid, 96% ethanol, hydrochloric acid (HCl), Isopropanol and Tris base were from Sigma Aldrich.

#### 2.1.4 CYP3A4 mRNA gene expression assay

Oligonucleotide primers were custom-synthesized by Invitrogen Life Technologies and Sigma Aldrich. TRIzol^®^ (Total RNA Isolation Reagent), Oligo (dT) 12–18 primers, M-MLV Reverse Transcriptase, RNAseOUT, DNAse I Amplification Grade and 100 mM dNTP Set were purchased from Invitrogen Life Technologies. SYBR Green (2xqPCR Master Mix premixed with SYBRgreen) was obtained from Amethyst reagents (Cambridge Bioscience). Rifampicin and DMSO were purchased from Sigma Aldrich.

#### 2.1.5 Human pregnane X receptor assay

Lipofectmamine^®^ 2000 was purchased from Invitrogen. Rifampicin and Luciferase assay system were obtained from Sigma Aldrich and Promega, respectively.

#### 2.1.6 Intracellular glutathione assay

Buthionine sulfoximine (BSO), L-glutathione reduced, Glutathione reductase, 5-5′-dithiobis (2-nitrobenzoic acid) (DTNB), β-Nicotinamide adenine dinucleotide 2′-phosphate reduced tetrasodium salt hydrate (NADPH), Triton-X and sulfosalicylic acid were from Sigma Aldrich.

#### 2.1.7 Gamma-glutamyl transferase activity assay

L-Glutamic acid γ-(p-nitroanilide) hydrochloride, 4-nitroaniline, Glycyl-glycine (Gly-Gly), Tris base, and acivicin were from Sigma Aldrich.

#### 2.1.8 Rhodamine 123 uptake assay

Rhodamine 123 was from Sigma Aldrich. Verapamil (Securon IV 2.5 mg/ml) was from Abbott Laboratories Ltd.

### 2.2 Plant materials and extraction

Pharmaceutical grade herbal materials for use in *Farmacias Vivas* (“Living Pharmacies,” i.e., pharmacies authorised to dispense herbal drug preparations made within the premises) (BRASIL, 2010, BRASIL, 2013) were kindly donated by Santos Flora Comércio de Ervas (Mairiporã, SP, Brazil). All handling and extraction were performed at UnB Faculty of Health Sciences. Herbal drugs (100 g) were subjected to a 20 min infusion (1:10) to mimic traditional use. The infusions were filtered (Whatman, Millipore, Ireland), lyophilized, and samples stored at −18°C throughout the studies both at UnB and UCL. Extracts were reconstituted in sterile distilled water for the bioassays.

Special permission must be granted from the Brazilian Council for Management of Genetic Heritage (CGEN) in order to have access to genetic material, respecting international intellectual and genetic property rights laws. Access was authorized (license no. 010295/2014-3) resulting from a collaboration between University College London (UCL) and UnB. Access was granted in May 2014, and the herbal drug preparations were received in June 2014. Moreover, this research was registered at the Brazilian National System for Management of Genetic Heritage (SisGen), under the number A71601C.

### 2.3 HPLC analysis

HPLC-UV analysis: Equipment consisted of an Agilent 1200 series HPLC system with a UV-VIS PDA detector (Agilent Technologies, United States), Agilent ChemStation software, Phenomenex^®^ C18 column (250 mm × 4.6 mm id, 5 μm). Solvent A (H_2_O + acetic acid 0.2% v/v) and B (methanol + acetic acid 0.2% v/v) were mixed in gradient mode as follows: 0 min 90% A, 0–5 min 80% A, 5–65 min 50% A, 65–75 min 20% A; flow rate 0.8 ml/min. The injection volume and column temperature were set at 10 μl and 40°C, respectively.

### 2.4 Cell culture

HepG2 cells were cultured in Minimal Essential Medium (MEM) Alpha supplemented with 10% fetal bovine serum (FBS), 100 U/ml penicillin, 100 μg/ml streptomycin. Caco-2 cells were cultured in Dulbecco’s Minimum Essential Medium (DMEM) with high glucose (4.5 g/L), and L-glutamine supplemented with 10% FBS, 100 U/ml penicillin, 100 μg/ml streptomycin, 1% non-essential amino acids (NEEA) and Vincristine (50 μM). HeLa cells were cultured in DMEM media were supplemented with 10% FBS, 100 U/ml penicillin, 100 μg/ml streptomycin. All cell lines were kept in the NuAire DH Autoflow CO_2_ Air-Jacketed incubator at 37°C/5% CO_2_.

### 2.5 Cell viability assays

The SRB, NR, and MTT assays were performed as previously described (Ruiz et al., 2006; Houghton et al., 2007; Repetto et al., 2008).

For the SRB assay, after 24 h incubation of HepG2 cells (2 × 104 cells/well) with the herbal drug extracts (100 μg/ml), media containing the samples was removed, and cells were fixed with 100 µl of cold 40% w/v TCA solution in deionized water. The plates were incubated at 4°C for 1 h and then immersed five times in tap water. The TCA-fixed cells were stained by adding 100 µl of SRB solution (0.4% SRB in 0.1% glacial acetic acid) and left at room temperature for 1 h. Afterward, the plates were quickly rinsed four times with 1% acetic acid and flicked to remove the unbound dye and then left to air-dry overnight. After drying completely, the protein bound SRB was solubilized by adding 100 µl of Tris base buffer solution (10 mM) to each well. The plates were agitated in an orbital shaker for 30 min. The optical density was measured at 492 nm by using a microplate plate reader Infinite M200 (Tecan Trading AG, Switzerland).

For NR, after 24 h incubation of Caco-2 VCR cells (1 × 10^4^ cells/well) with the herbal drug extracts (100 μg/ml), media was removed and 100 µl of NR solution (40 μg/ml) pre-warmed at 37°C was added to each well and all plates were placed in the incubator at 37°C for 2 h. The cells were rinsed with 150 µl of PBS, and the washing solution removed by decanting or gently tapping the plate. 150 µl of neutral red destain solution (96% ethanol, deionised water, glacial acetic acid; 50%:49%:1%) was added to each well of the plate after the washing step. The plate was immediately shaken on a microplate shaker (IKA MS3, Germany) for at least 10 min until the neutral red had been completely extracted from the cells and formed a homogenous solution. The absorbance of the neutral red extract was read out in the plate reader at 540 nm.

For MTT assay, after 24 h incubation of HeLa cells (2 × 10^4^ cells/well) with the herbal drug extracts (100 μg/ml), media containing the samples was removed, and 200 µl of fresh media containing 50 µl of MTT (1 mg/ml) was added to each well. After 4 h incubation, the solution was carefully aspirated and replaced by 150 µl of MTT solubilization solution (0.04 M HCl in isopropanol). Plates were shaken until crystals were utterly dissolved and absorbance was measured at 570 nm in the plate reader.

### 2.6 Real-time RT-qPCR analysis

#### 2.6.1 mRNA extraction and cDNA synthesis

After exposing HepG2 cells (5 × 10^5^ cells/well) to the herbal drug extract or the CYP3A4 inducer Rifampicin (50 mM) or the CYP3A4 inhibitor DMSO 1% for 96 h, total RNA was extracted from using TRIzol^®^ Reagent according to the manufacturer’s protocol. Samples were treated with DNase I (1 U/μl) to avoid genomic contamination. The quantity and the quality of RNA were determined by differential readings at 260 and 280 nm in a Nanodrop 2000 (Thermo Scientific). The integrity of total RNA from HepG2 cells was assessed by visual inspection of the two rRNAs 28 and 18 s on agarose gels. cDNA was synthesized from 1 μg of total RNA with the Moloney rine Leukemia Virus Reverse Transcriptase (M-MLV RT) (200 U/μl) and oligo (dT) 12–18 primer (0.5 μg/μl), according to the manufacturer’s instruction in a final volume of 21 μl.

#### 2.6.2 RT-qPCR conditions and analysis

CYP3A4 sense strand primer sequence was 5′-CAA​GGA​CAA​CAT​AGA​TCG​TTA​CAT​ATA​CAC​ACC​CTT​TGG​AAG-3′ and the antisense strand primer was 5′-AGC​TCA​ATG​CAT​GTA​CAG​AAT​CCC​CGG​TTA-3′. The β-actin gene was used to control for variations in RNA loading within the experimental conditions. The sense strand primer sequence was 5′-CGT​ACC​ACT​GGC​ATC​GTG​AT-3′ and the antisense strand primer was 5′-GTG​TTG​GCG​TAC​AGG​TCT​TTG-3′. The RT-qPCR was carried out in 96-well plates using a Pikoreal™ Real-Time PCR detection system (Thermo Scientific). Each well contained a final reaction volume of 10 μl (5.0 μl MasterMix with SYBR Green, 2.0 μl cDNA template diluted appropriately, 0.5 μl of each primer at a final concentration 0.3 mM and 2.0 μl of RNAse/DNAse free distilled water). PCR reaction was performed under the following schema: Initial denaturation at 95°C for 2 min, then 40 cycles of denaturation at 95°C for 15 s, annealing at 55°C (β-actin) or 60°C (CYP3A4) for 30 s, and extension at 72°C for 30 s.

At the end of the run, a melting curve was generated by heating the amplicon from 60°C to 95°C in order to confirm the specificity of the amplification for each primer pair. All RT-qPCR were run in quadruplicates. Standard curves were produced to check the PCR efficiency using a five-fold dilution series of cDNA. Efficiency (E) of primer pairs was obtained from the slope of the calibration curve generated. The relative expression was calculated based on “delta delta Ct” (ΔΔCt) values. Normalization of the target gene was achieved by using β-actin as a reference gene.

### 2.7 Human pregnane X receptor activation assay

After 24 h seeding, HeLa cells (4 × 104 cells/well) were transiently co-transfected with 60 ng of pM-Gal4-PXR-LBD and 240 ng of Gal4 luciferase reporter using lipofectamine 2000 reagent according to the manufacturer’s protocol. Transfected cells were treated with increasing concentrations of herbal drug extract and/or rifampicin 1 μM (EC_50_) as a positive control. Luciferase activity was measured after 24 h, according to manufacturer’s protocol in a 20/20n Glomax luminometer and reported as response (%) compared to cells treated only with vehicle.

### 2.8 Intracellular glutathione levels

The method used in the intracellular determination of glutathione levels was adapted from those described by Rahman and coworkers (Rahman et al., 2006), and Allen and coworkers (Allen et al., 2000) with slight modifications. After 24 h incubation with BSO (10 μM) or herbal drug extract (100 μg/ml), HepG2 cells (4 × 10^4^ cells/well) were washed with PBS and 60 µl of 0.1% Triton-X was added to each well of the plates to lyse the cells. 25 µl of 5% sulfosalicylic acid was added to the cell lysates, and plates were shaken for 2 min. 25 µl of glutathione reaction buffer containing NADPH (2.39 mM), DTNB (0.01 M) and glutathione reductase (500 UI) in sodium phosphate buffer (143 mM) containing EDTA (6.3 mM) was added to the cell lysates. Absorbance was read in a kinetic cycle in the plate reader every 30 s for 5 min at 405 nm (11 readings). Absorbances were converted into absolute amounts through the i-slopes method using known concentrations of L-glutathione reduced.

### 2.9 Gamma-glutamyl transferase activity assay

GGT activity assay was conducted according to Rebbeor and coworkers (Rebbeor et al., 1998). Briefly, after 24 h incubation of HepG2 cells (1 × 106 cells/well) with the GGT inhibitor acivicin (5 µM) or herbal drug extract (100 μg/ml), media was aspirated, and cells were washed with PBS. 4 ml of pre-warmed glycylglicine buffer (115 mM Tris, 138 mM glycylglycine) and 400 µl of the substrate γ-Glutamyl-p-nitroanilide (29.6 mg/ml of HCl 0.5 mmol/L) were added to the wells and plates were incubated for 10 min. Then, 500 µl of the content of each well were transferred to 24-well plates and absorbance was measured in the plate reader (405 nm). Absorbances were converted into absolute amounts by means of a calibration line using 4-nitroaniline.

### 2.10 Rhodamine 123 uptake assay

Rhodamine uptake/efflux assays were conducted as described by Chieli and coworkers with minor modifications (Chieli et al., 1993). After five passages in media containing vincristine (50 µM), Caco-2 VCR cells (1 × 104 cells/well) were incubated for 2 h with the P-gp inhibitor verapamil (20 µM) or herbal drug extract (100 μg/ml) in serum-free media containing rhodamine 123 (5 μg/ml). After incubation, cells were washed with verapamil (20 µM) in PBS. Cells were lysed with 100 µl of 0.1% Triton X-100 in PBS, and the plates were placed in the incubator for 15 min. The fluorescence intensity of cell lysates was measured in the plate reader (Exc-485nm, Em-525 nm). The cellular accumulation of rhodamine 123 for each of the extracts was expressed as the percentage of the accumulation measured for rhodamine 123 under control conditions.

### 2.11 Statistical analysis

Collected data were analysed as means ± SEM of at least three independent experiments. Statistical significance was measured by Student’s *t*-test, and ANOVA followed by Bonferroni correction using GraphPad InStat (GraphPad Software Inc., CA, United States). Results with a value of *p* < 0.05 were considered significant.

## 3. Results and discussion

### 3.1 In vitro effects of herbal preparations on the selected experimental models

For the first time, we report the overall effects/activity of clinically relevant herbal preparations from both the Latin American and European traditions on important preclinical targets for drug metabolism. To facilitate its interpretation, all results are summarised in Table 3.

**TABLE 3:** Summary of the effects/activity of the selected herbal preparations on the studied pharmacokinetic targets. This “Heat map” is based on the significance of either the inhibition (red shades) or enhancement (green shades) of the measured endpoint for each herbal preparation according to Bonferroni Post-ANOVA test.

	CYP3A4 expression	Glutathione Level	GGT activity	P-gp Efflux
*A. absinthium*	0	0	0	0
*B. pilosa*	0	−3	0	0
*C. sylvestris*	−1	−3	0	0
*C. spicatus*	0	−2	−1	0
*E. arvense*	−1	−2	0	0
*F. purshiana*	−1	−3	3	0
*H. impetiginosus*	0	−3	0	0
*L. album*	1	−3	0	0
*L. ferrea*	−3	−3	3	−3
*M. sylvestris*	0	−1	0	0
*M. ilicifolia*	−2	−3	0	0
*M. nigra*	0	0	0	0
*P. incarnata*	−2	−2	−1	0
*P. americana*	−2	−3	−2	0
*S. alba*	−3	−2	−2	−3
*S. terebinthifolia*	−3	−3	−1	−3
*S. chilensis*	0	−2	0	0
*S. cumini*	−3	0	−2	−3
*V. phosphorica*	−3	−3	0	0

**FIGURE 1:**
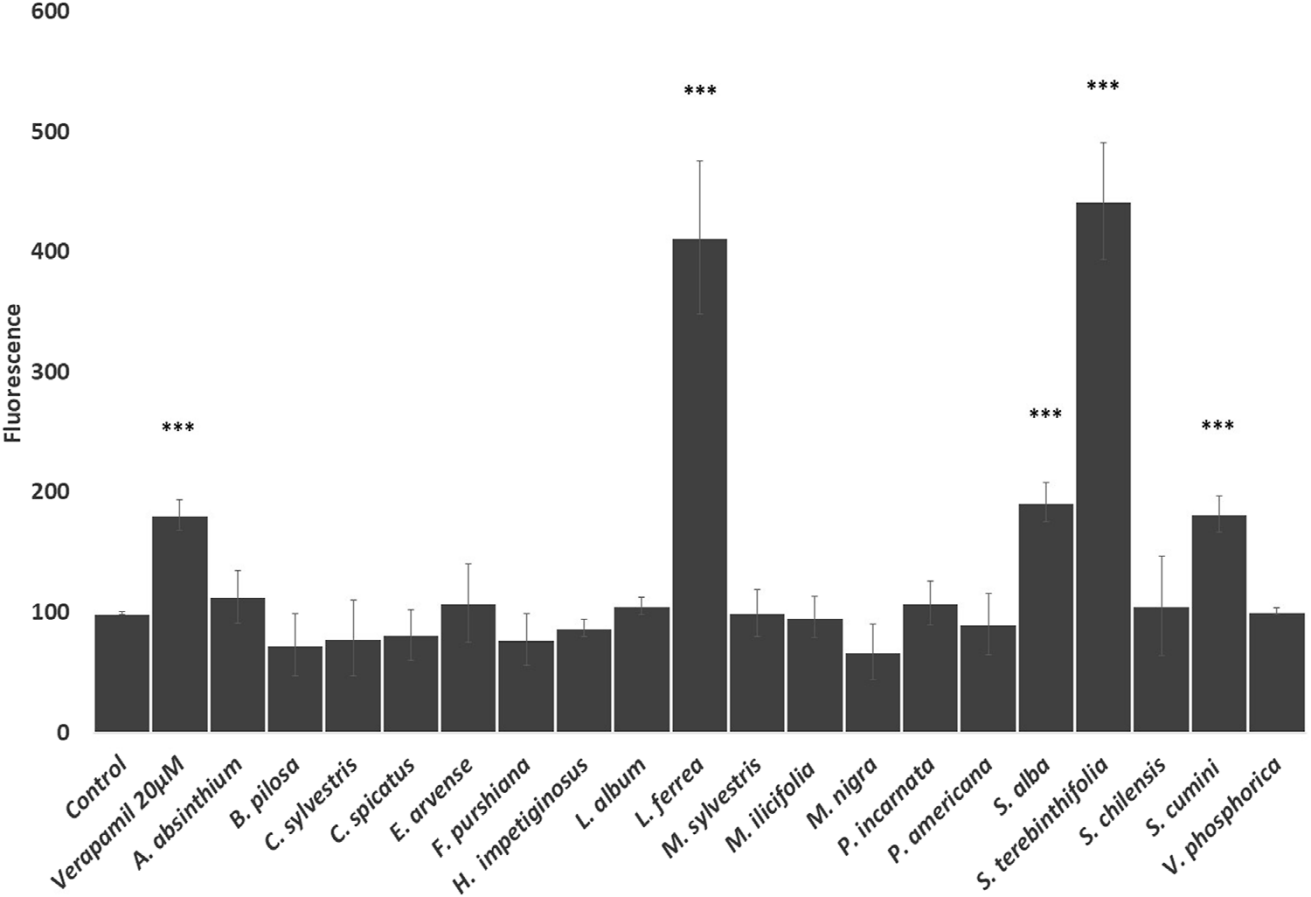
Effects of the selected herbal preparations (100 μg/ml, except *S. alba* 10 μg/ml) and Verapamil (positive reference treatment) on the efflux activity of P-gp in CaCo2 VCR cells (Mean ± SEM, n ≥ 3). Statistical significance against water (control) according to Bonferroni (*p < 0.05, **p < 0.01, ***p < 0.001).

The *in vitro* effects of the traditional herbal preparations (i.e., aqueous extracts or infusions which HPLC fingerprints are provided as Supplementary Figures) (see Supplementary Materials) on P-gp activity were evaluated by measuring the rhodamine uptake/efflux in Caco-2 VCR cells (Figure 1). As we can observe, rhodamine accumulation was significantly increased when the cells were treated with extracts from Latin American tradition such as *L. ferrea*, *S. terebinthifolia*, and *S. cumini*, demonstrating that these herbal drug extracts strongly inhibited rhodamine efflux (Figure 1). Among the European traditional herbal preparations only *S. alba* inhibits rhodamine efflux. Accordingly, it is conceivable that the coadministration of these herbal extracts with medicines/drugs acting as substrates of P-gp may change the bioavailability of these drugs. The clinical relevance of such interaction would need to be confirmed through future clinical studies.

**FIGURE 2:**
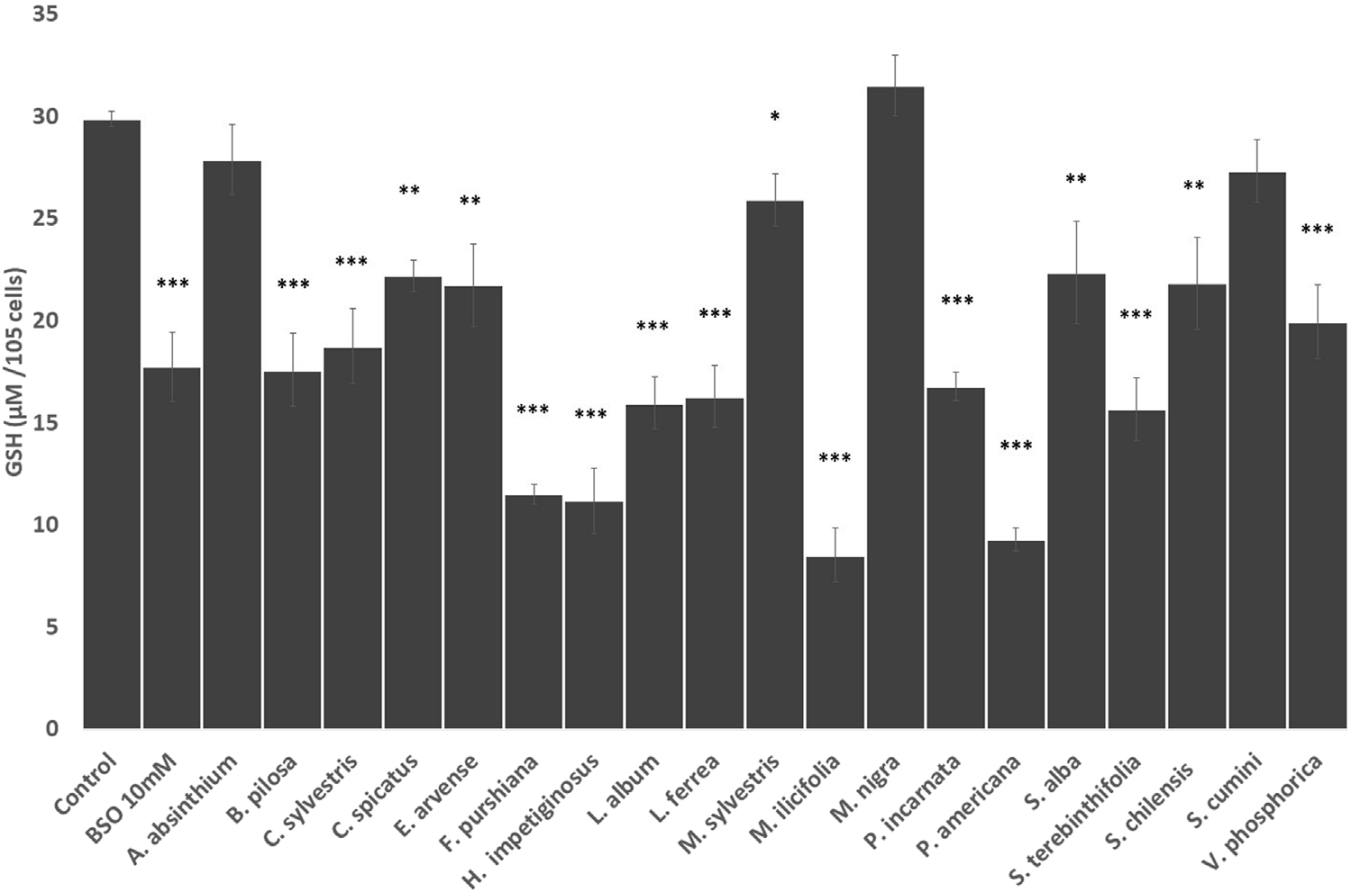
Effects of the selected herbal preparations (100 mg/ml) and buthioninesulphoximine (BSO, 10 mM, positive reference treatment) on the levels of intracellular glutathione in HEPG2 cells (Mean ± SEM, *n* ≥ 3). Statistical significance against water (control) according to Bonferroni (*p < 0.05, **p < 0.01, ***p < 0.001).

Next, we evaluated the influence of the herbal drug extracts on the intracellular concentration of glutathione in HepG2 cells. As shown in Figure 2, all the Latin American herbal preparations significantly decrease the intracellular concentration of glutathione in HepG2 cells at comparable levels to that produced by the positive control buthionine sulfoximide except *S. cumini*. Similarly, all European herbal preparations significantly decrease the intracellular concentration of glutathione except *A. absinthium and M. nigra* (Figure 2).

**FIGURE 3:**
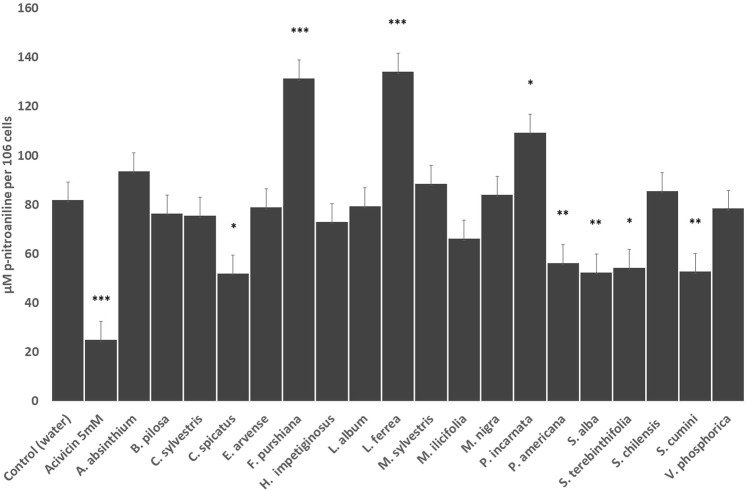
Effects of the selected herbal preparations (100 μg/ml) and Acivicin (5 μM, positive reference treatment) on the surface enzymatic activity of GGT of HEPG2 cells (Mean ± SEM, *n* ≥ 3). Statistical significance against water (control) according to Bonferroni (*p < 0.05, **p < 0.01, ***p < 0.001).

To examine whether the glutathione depletion may be mediated by inhibition of the Gamma-Glutamyl transferase (GGT) activity, we measured the effects of the herbal preparations by the amount of p-nitroaniline liberated by the surface transferase reaction in HepG2 cells (Figure 3). We demonstrated that 4 of the 12 Latin American herbal drug extracts (*C. spicatus, P. americana, S. terebinthifolia* and *S. cumini*) significantly reduced GGT activity similarly to the positive reference drug acivicin, and one of them (*L. ferrea*) greatly increased the enzyme activity (Figure 3). Among the European herbal extracts, only *S. alba* preparation was able to impair GGT activity whilst *P. incarnata* and *F. purshiana* increased the activity of the enzyme (Figure 3).

Glutathione is key for the clearance of drugs and the deactivation of reactive species (Xie, 2009). Its intracellular levels may be affected by several mechanisms. The most obvious is the inhibition and/or induction of enzymes involved in its biosynthesis (Lu, 2013). In this regard, the presence of some compounds in the extracts could be depleting GSH in a similar way to buthionine sulfoximine, that is, by inhibiting the GGT enzyme (Oguchi et al., 1994) or just inhibiting glutamate-cysteine ligase (GCL) that is responsible for the first and rate-limiting step in GSH synthesis (Franklin et al., 2009). To identify these active compounds, either dereplication or bioguided isolation, would be needed. Literature data report that polyphenols such as gallic acid and derivatives and certain flavonoids induce GCL expression rather than inhibiting its activity (Panich et al., 2012; Huang et al., 2013). All herbal preparations lowered intracellular levels of glutathione except for *A. absinthium*, *M. nigra*, and *S. cumini*. Gallic acid has been identified as a prominent compound in these very same herbal drug preparations so in our hands its presence did not induce any noticeable *in vitro* effects.

The reduction of GSH levels in HepG2 cells treated with *C. spicatus, P. americana, S. alba, S. terebinthifolia* and *S. cumini* turned out to be due—at least in part- by their ability to lower GGT activity. However, *F. purshiana*, *L. ferrea and P. incarnata* seem to stimulate its *in vitro* activity, whilst the remaining herbal preparations did not affect GGT activity in a significant way, so GSH levels in all those cases may have been affected *via* other mechanisms.

We attempted the dereplication of potential GGT inhibitors using HPLC-UV analysis. Then again gallic acid has been reported to deplete intracellular GSH levels in melanoma cells through inhibition of the activity of the Gamma-Glutamyl-cysteine synthetase (Locatelli et al., 2009). Gallic acid has also been proven to inhibit GGT activity in mice (Mahajan and Mahmood, 2009). Therefore, the GGT inhibition brought about by two of the herbal preparations here studied (*S. terebinthifolia* and *S. cumini*) may be explained on this basis and further studies are needed to confirm this point.

More refined experiments would be needed to attest the formation of GSH-adducts which could cause a reduction of the detectable intracellular GSH without compensation by *de novo* GSH (Blair, 2010), and/or modulation of GST activity (Tolson and Wang, 2010). The modification of GSH efflux from cells may also be associated with cell death (De Nicola and Ghibelli, 2014) but this is not likely happening as were working with concentrations of herbal drug extract more than ten times lower than their maximum non-toxic concentration (>1,000 μg/ml).

**FIGURE 4:**
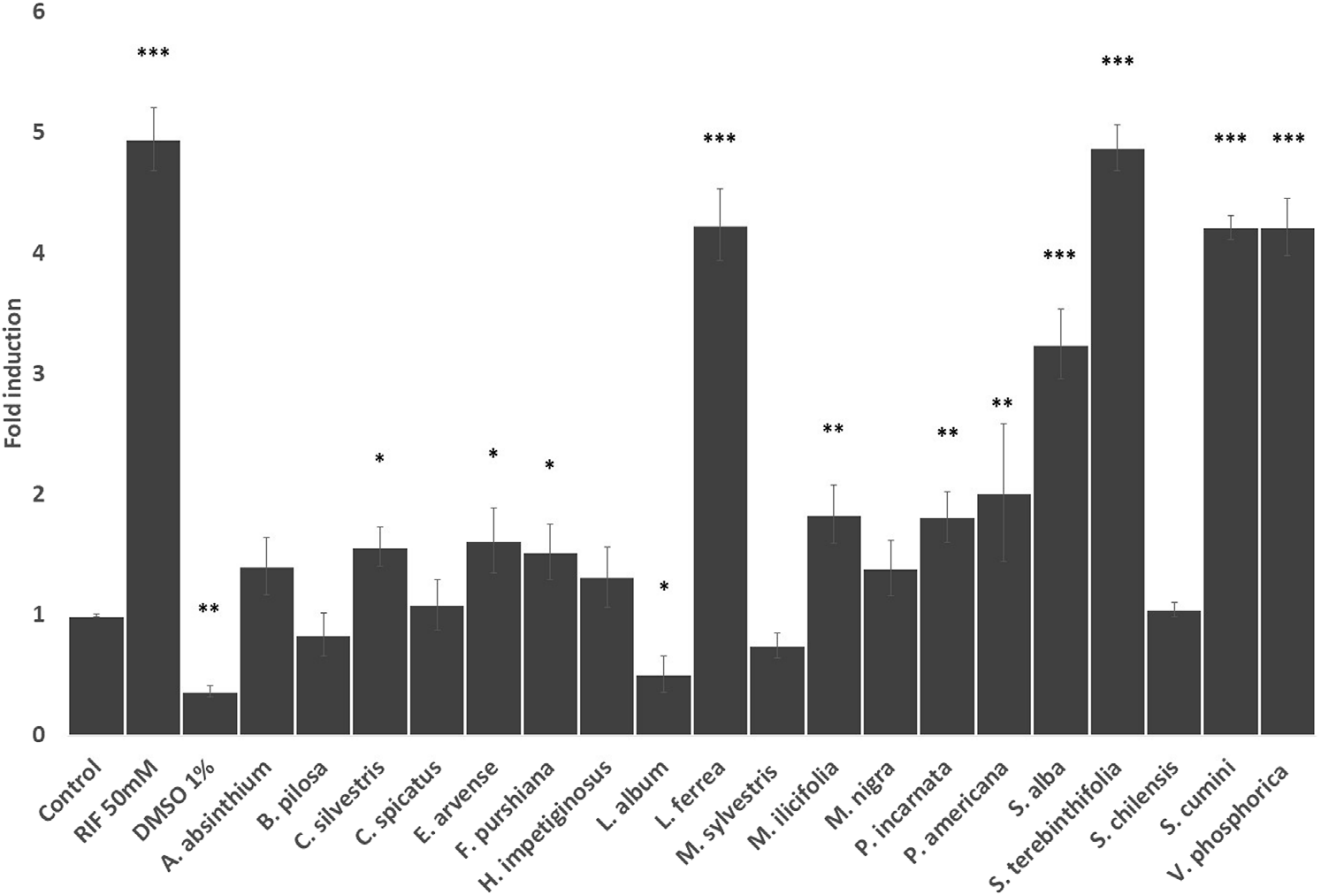
Effects of the selected herbal preparations (100 μg/ml) and Rifampicin (RIF, 50 mM, positive reference) on the expression of CYP 3A4 in HEPG2 cells (Mean ± SEM, *n* ≥ 3). Statistical significance against DMSO 0.2% (control) according to Bonferroni (*p < 0.05, **p < 0.01, ***p < 0.001).

To further investigate if the herbal drug extracts modulate CYP 3A4 expression levels in HEPG2 cells we performed RT-qPCR analysis. As seen in Figure 4, extracts from Latin American plants such as *L. ferrea, S. terebinthifolia, S. cumini,* and *V. phosphorica* induced a similar increase in CYP3A4 expression to the one exerted by rifampicin (*p<0.001*), which strongly enhanced CYP3A4 expression by 5-fold. Other herbal preparations that enhanced CY3A4 expression but to a less extend were *C. sylvestris*, *M. ilicifolia*, and *P. americana*. Among the European plants, only *S. alba* increased CYP3A4 expression levels in HEPG2 cells as strongly as rifampicin with *E. arvense*, *P. incarnata* and *F. purshiana* also enhancing CYP3A4 expression to a lower extend. Conversely, *L. album* reduced the CYP 3A4 gene expression (Figure 4).

Recent research suggested that *M. ilicifolia* extracts inhibit the intestinal metabolism and transport of drugs mediated by CYP3A and P-gp, respectively (Nascimento et al., 2020). In our hands the P-gp activity in CaCo2 cells was not affected and in HepG2 cells it stimulates the expression of the CYP3A4 gene, so the effects of these medicinal plant warrant further research.

**FIGURE 5:**
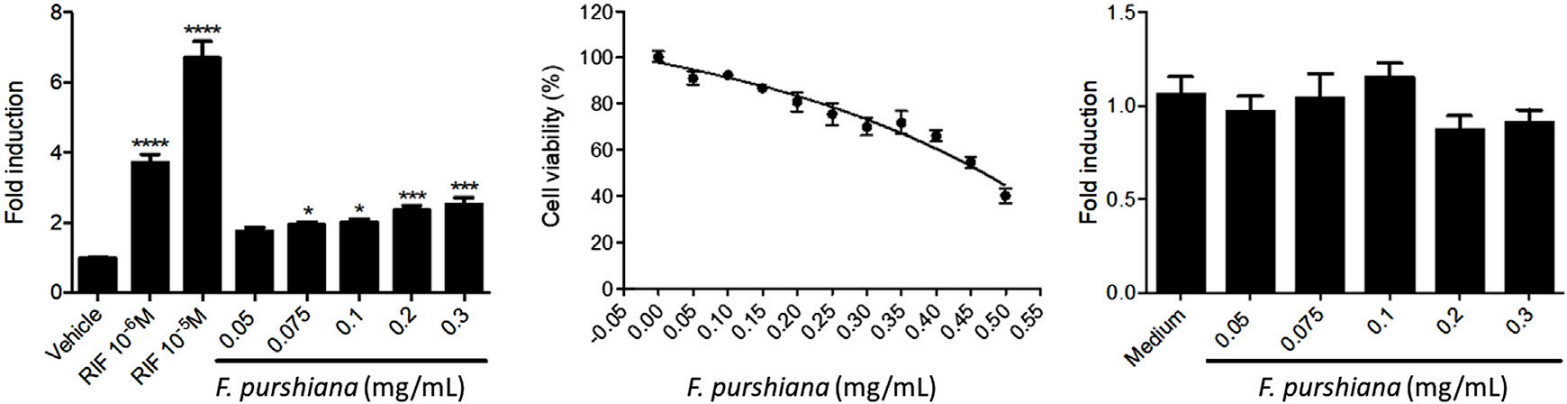
Effects of *Frangula purshiana* on (left) the activation of hPXR in transiently transfected HeLa cells and Rifampicin (RIF, positive reference), (centre) cell viability in the same cell line and (right) expression vector pCMV luciferase reporter in co-transfected HeLa cells. (Mean ± SEM, *n* ≥ 3 Statistical significance against DMSO 0.2% (control) according to Bonferroni (*p < 0.05, **p < 0.01, ***p < 0.001).

To evaluate whether the activation of hPXR may be mediating the modulation of CYP3A4 gene expression, HeLa cells were co-transfected with expression vector pM-Gal4-PXR-LBD and Gal4 luciferase reporter and treated with increasing concentrations of herbal preparations. Our results show that none of the Latin American herbal extracts that significantly increased CYP3A4 expression activate PXR. Only the aqueous extract of *F. purshiana* showed a dose-dependent activation of hPXR (Figure 5). Of note, the aqueous extract of *L. album* also seemed to activate hPXR in transiently transfected HeLa cells at 0.3–0.5 mg/ml. However, it also activated the pCMV luciferase reporter and slightly stimulated cell proliferation (Data not shown). Therefore, *L. album* may not be a true PXR agonist, and the observed increase in the transcriptional activity of hPXR may be due to a greater number of cells and/or interference with the CMV promoter that drivers the expression of hPXR. The mechanism to explain why some plant extracts induced the CYP3A4 expression without stimulating hPXR is unknown. It is conceivable that these plants modulate other transcription mechanisms involved in regulating the CYP3A4 promoter that is PXR independent. A similar result was previously described by compounds isolated from the roots of *Euphorbia fischeriana* Steud. Some of the ligands isolated in the root increase CYP3A4 expression by stimulating the transcription activity of the CYP3A4 promoter in the region regulated by PXR (−7832 to −7599—xenobiotic responsive enhancer module—XREM regulatory region of CYP3A4). On the other hand, other compounds stimulate the proximal region of the CYP3A4 promoter, which is PXR independent (Kuang et al., 2014). Moreover, constitutive androstane receptor (CAR), and vitamin D receptor (VDR) are also capable of binding in the same regulating cis-acting elements as PXR, promoting a crosstalk between these signalling pathways modulate the xenobiotic response (Pascussi et al., 2003). Also, it has been shown that the hepatocyte nuclear factor-3 (HNF-3), HNF-4, and CCAAT/enhancer-binding protein (C/EBP), nuclear factor I (NFIA1.1, NFIB2, NFIC1, NFIC2, and NFIX) play important roles in regulating the expression CYP3A4 (Rodriguez-Antona et al., 2003; Riffel et al., 2009). Additionally, some compounds can regulate the expression of CYP3A4 by modulating the histone methylation in the promoter and enhancer regions of CYP3A4 (Kouzarides, 2007).

#### 3.2 Contribution, choices, and limitations of this study

As highlighted in the introduction, the pharmacokinetic profile of complex herbal medicines is largely unknown except for several “high profile” cases such as St John’s Wort (*Hypericum perforatum* L.), garlic (*Allium sativa* L.) and ginkgo (*Ginko biloba* L.) among a few others, as they sparked the interest for the field of “herb—drug interactions” back in the 1970s–1980s (Silveira et al., 2018). Since, there has been little economic incentive for the industry to invest in studying in such aspects, as most of the regulatory requirements for herbal medicines are based in “well-stablished traditional use” which “waives” any preclinical and clinical studies for the commercialisation of medicinal herbal products (Silveira et al., 2018).

Therefore, any pharmacokinetic preclinical experimental data for herbal medicines are always important in that they deal with an “orphan” field of knowledge. We here present a series of original data on many herbal medicines, some of them with long standing use (such as *Salix alba* L.) to help shape future research. Our previous research in the field has been already used and cited by important institutions such as ANVISA (BRASIL, 2021) and WHO (WHO, 2021) as sources of useful preliminary data to inform both researchers and healthcare professionals. We expect this work to continue adding to the increased knowledge of the efficacy and safety of such herbal preparations.

Drug metabolism studies are an essential step in the preclinical development of medicines. To this end, *in vitro* models must provide vital information on the effects of the lead drug candidate (LDC) upon drug metabolising enzymes, such as the CYP, as a step towards predicting potential DDI. Many *in vitro* experimental models of drug metabolism and disposition make use of cells; human hepatocytes (HH) or their microsomes (HLM), for example, are considered the best models for enzyme inhibition, whereas HLM is the preferred test system for enzyme induction (Guo et al., 2011). Caco-2 cells are a common choice for transporter studies (Zhang et al., 2012). Several other models have been successfully developed to replace the use of cells and have been validated for predicting the effects of single drug entities in the metabolism with similar correlation to HLM. Such is the case of characterised metabolic recombinant enzymes (CYP1A2, 2C9, 2C19, 2D6, and 3A4) expressed in *Escherichia coli*, which has been developed by the pharmaceutical company AstraZeneca (McGinnity and Riley, 2001). The use of *in silico* models has become more common in the pharmaceutical industry in recent years to speed up the selection of the best lead candidates at this stage of drug development (Steinmetz and Spack, 2009).

To be accepted by regulatory bodies, drug metabolism models must be robust, validated and reproducible. However, unlike the requirements for synthetic drug studies, there is no regulatory requirement for preclinical metabolism studies of traditional herbal medicines and no substantial industrial activity exists on this side. Metabolism studies on traditional herbal medicines are normally only initiated upon the receipt of case reports documenting a supposed interaction, which trigger warnings from the regulators. Otherwise, such studies originate from purely academic-driven programmes focused on demonstrating the potential effects of traditional herbal medicines on metabolic and transporter targets (Brantley et al., 2014); these studies are generally carried out by adapting protocols to their own needs, thus leading to an array of different experimental approaches.

The established principles and models used for metabolism studies in synthetic drugs are directly applicable to herbal medicines (He et al., 2010). However, they would require significantly high budgets and infrastructure. Due to the scope of the academic activities, a case can thus be made regarding the choice of cell lines. HH are generally replaced by other cell models like HepG2 and HepaRG cells for determination of metabolic activities. The hepatoma cell line HepG2 is one of the most popular models for pharmacological and toxicological *in vitro* studies in academia despite having lost some liver functions, and its supply is readily available from any public cell bank (Guo et al., 2011). The HepaRG cell line is derived from another hepatocellular carcinoma cell and it maintains important metabolic activities lost in HepG2 cells. But it is significantly more expensive and only available through a private company and still has lost many functions when compared to HH (Szabo et al., 2013).

The high costs of HepaRG and HH cells forces many researchers to use the cheaper HepG2 cell line. A vial containing 2–3 × 10^6^ HepG2 cells costs around 330 GBP and stocks can be made for further use whenever they are needed. On the other hand, a vial containing 10 million HepaRG cells would cost nearly double at about 600 GBP, and cells would have to be plated out in 24-well plates for immediate use. Furthermore, HepG2 is an immortal cell line and therefore it is suitable for long-term maintenance. It generally reaches 70%–80% confluence within 48 h, which allows the conduction of medium and high throughput screenings. Long-term maintenance of HepaRG and HH with high availability, on the other hand, could be very challenging in traditional two-dimensional monolayer cultivation systems (Klein et al., 2014).

Caco-2 cells are one of the most used *in vitro* models for the assessment of P-gp transporter activity within most pharmaceutical industries (Eneroth et al., 2001) and reports of use of Caco-2 for transporter studies in herbal medicines have been also well documented (Yoshida et al., 2006; Li et al., 2014; Wu et al., 2016)**.** The reasons for choosing this cell model for efflux studies are like those that were applied to HepG2: The reduced costs and rapid availability for immediate use.

Conduction of CYP3A4 gene expression was made using RT-PCR, which is the most used technique for such a study. Due to the costs of RT-PCR reagents, it was necessary to focus our phase I metabolism study on the expression of CYP3A4 only as it is responsible for metabolising more than 50% of the currently marketed drugs. Despite the availability of several protocols for HH, the numerous steps and reagents used to produce the cDNA and to amplify the CYP3A4 gene had to be all optimised as this technique is highly dependent on the instrumentation and the cell line. In the future other researchers may want to screen the selected herbal preparations for activity upon other CYP isoforms.

The studies found in the literature made use of different concentrations of the gene inducer, total RNA and time of the treatment to achieve significant levels of CYP3A4 expression with different cell lines. For instance, Li and coworkers used RT-PCR for determination of the CYP expression in HepG2 cells by using 1 µg of total RNA. The amount of RIF used to achieve 2.5 fold induction was 10 µM for 48 h treatment (Li et al., 2013). Modarai and coworkers, on the other hand, needed to use 2.5 µg of total RNA and RIF 50 µM treatment over a period of 96 h of exposition to achieve 3.83-fold induction also in HepG2 cells (Modarai et al., 2011). Lau and coworkers conducted research on the effects of ethanol extracts of the Chinese herbs *Oldenlandia diffusa* (Willd.) Roxb. (Rubiaceae)*, Codonopsis tangshen* Oliv. (Campanulaceae)*, Rehmannia glutinosa* (Gaertn.) DC. (Plantaginaceae) and *Astragalus propinquus* Schischkin (Leguminosae) on CYP3A4 expression using human colon adenocarcinoma-derived LS180 cells. In that experiment, the amount of RNA used for cDNA synthesis was not disclosed but a 24 h treatment with RIF 10 µM was reportedly enough to achieve 8-fold induction (Lau et al., 2013). Likewise, Liu et al. (2012) did not indicate the amount of total RNA used but their treatment of the colon adenocarcinoma LS174T cells with RIF 20 µM for 24 h resulted in 3-fold induction.

Contrary to a study using RIF 10 µM for 48 h conducted by Li et al. (2013), the treatment of our HepG2 cells did not significantly change the CYP3A4 expression using 1 µg of total RNA. In order to increase CYP3A4 expression using this inducer, some researchers may decide to increase the amount of total RNA amplified, as was the case in Modarai et al. (2011). However, we managed to achieve almost 5-fold induction by treating our HepG2 cells using the same RIF concentration for the same time treatment as Modarai et al. (2011) and the same total RNA concentration used by Li et al. (2013).

The screening of *in vitro* P-gp activity with Caco-2 cells is not devoid of drawbacks. One is the reported downregulation of the P-gp expression during cell culturing process. In addition, the P-gp expression is quite variable in this cell line which seems to be correlated with its origin and passage number (Shirasaka et al., 2008). In order to overcome these issues, several authors have proposed new ways to induce P-gp expression in Caco-2 cells. For instance, Eneroth and coworkers overexpressed P-gp in Caco-2 cells prior to applying their use in a calcein AM extrusion screening assay. To do so, they cultured normal Caco-2 cells in media containing several concentrations of vincristine. The group concluded that an optimal level of P-gp was achieved using vincristine 25 nM and that it would be suitable for their calcein AM extrusion method (Eneroth et al., 2001). Shirasaka and coworkers managed to increase the expression of P-gp by culturing Caco-2 cells in media containing vinblastine 50 nM. The mRNA concentration of P-gp increased in more than 4-fold compared to normal Caco-2 cells. This group proposed some possible reasons for the overexpression of P-gp in Caco-2 cells treated with vinca alkaloids. One possibility was that the overexpression of P-gp was mediated by the activation of hPXR. Another proposed explanation was that P-gp expression in Caco-2 is not uniform and that the vinca alkaloids treatment would have probably killed the cells with low P-gp expression, therefore acting as a selection process instead of induction. No conclusion has been reached about the correct P-gp overexpression mechanism yet (Shirasaka et al., 2006; Shirasaka et al., 2008). To complicate matters further, studies on P-gp efflux inhibition may use different substrates. A commonly used substrate probe is digoxin, however concerns have been raised about the use of this drug due to the high variability in the results. (Jouan et al. (2016) carried out an *in vitro* evaluation of P-gp efflux inhibition comparing Rh-123 vs. digoxin transport. The authors concluded that Rh-123 is the most convenient substrate for characterising the effects of several P-gp inhibitors, including verapamil. Importantly, the inter-laboratory reproducibility of digoxin transport experiments was lower when compared to the results found using Rh-123. Therefore, the predictability of drug interactions could be questioned using this substrate.

Thus, in our study we followed the protocol involving the overexpression of P-gp as reported by Eneroth et al. (2001). This resulted in an increased P-gp activity of Caco-2 cells cultured with vincristine compared to normal Caco-2, which made the conduction of our preclinical study possible. Despite this, the IC_50_ of verapamil–a reference for P-gp inhibition–in normal Caco-2 cells is not significantly different than in overexpressing Caco-2 cells after vincristine treatment (Mazzari et al., 2016).

These considerations involving cell models, suitable techniques and methodologies were crucial for the execution of the present study. Although most models and techniques used in the private sector are still out of reach of the average academic group, researchers in academia have been applying cutting-edge techniques to overcome the limitations of less sensitive models, thus making such studies accessible to all levels of research investment. This is probably one of the reasons why research on HDI has considerably improved over the years, even as funding is becoming very limited in many countries. The *in vitro* assessment of the effects of herbal medicines in metabolic and transporter targets is an important step for prediction of HDI. Our findings and the influence of intrinsic factors in the metabolism are limited by our choices and other researchers may take advantage by testing in more clinically relevant and elaborated models the herbal preparations that we have shown to be active in our screening approach.

## 4 Conclusion

The present preclinical study provides for first time evidence that some herbal preparations from the Latin American and European phytotherapy traditions catalogued by the Brazilian Unified Health System as herbal medicines of interest for the Brazilian population cause *in vitro* disturbances to metabolic mechanisms.

On the one side, two herbal preparations (*A. absinthium* L. and *M. nigra* L.) did not show significant *in vitro* effects on any of the studied pharmacokinetic targets. Although these results cannot discard any potential *in vivo* effects they may imply a safer margin of use within the limitations of this study.

On the opposite extreme, *Libidibia ferrea* (Mart.) L. P. Queiroz, *Frangula purshiana* Cooper, *Schinus terebinthifolia* Raddi, and *Salix alba* L. were significantly modulating the *in vitro* function of all targets. These medicinal plants will be prioritised for further *in vivo* studies using more refined preclinical methods. In the meantime, we recommend a more active pharmacovigilance approach for these herbal preparations.

The rest of herbal preparations present a mix of *in vitro* activities that may merit more focused approaches depending on the researcher’s interest in one or another of the ADME aspects. It is not possible to make any clinical predictions from these data as they are of interest for the basic experimental scientist only. Moreover, the complexity of the chemical composition and variability of the herbal preparations here studied requires that similar work is undertaken in other regions with the same herbal drugs to map their potential ADME effects upon different populations.

It is important to stress that the traditional use for all the herbal drugs here studied has been well stablished for many Medicines Agencies. This implies that they are endowed with a broad safety baseline within the doses and indications established by the relevant regulatory body. As always, patients and public alike are reminded that taking drugs and herbal preparations together without consulting with a knowledgeable healthcare professional -particularly when a hospital intervention has been scheduled- may pose serious risks.

## Data Availability

The original contributions presented in the study are included in the article/Supplementary Material, further inquiries can be directed to the corresponding authors.
