# Supplementary material for: In vitro effects of European and Latin-American medicinal plants in CYP3A4 gene expression, glutathione levels, and P-glycoprotein activity
Source: Front Pharmacol. 2022 Oct 5;13:826395. doi: 10.3389/fphar.2022.826395 (PMC9579425; doi:10.3389/fphar.2022.826395)
Supplement: Supplementary file 1 [file Table1.DOCX]

Supplementary Material

*In vitro* effects of European and Latin-American medicinal plants in CYP3A4 gene expression, glutathione levels and P-glycoprotein activity.

Andre L. D. A. Mazzari^1^, Mariella Lacerda^2^, Flora Milton^2,3^, João Augusto Mulin Montechiari Machado^3^, Simone Batista Pires Sinoti^2^, Anne-Soulene Toullec^1^, Patricia Marques Rodrigues^2^, Luiz Alberto Simeoni^2^, Francisco A.R. Neves^2,*^, Dâmaris Silveira^2,*^, Jose M. Prieto^1,2,4,*^.

^1^ School of Pharmacy, University College London, London, United Kingdom.

^2^ Faculdade de Ciências da Saúde, Universidade de Brasília - Brasília, Distrito Federal, Brazil.

^3^Instituto de Saúde de Nova Friburgo, Universidade Federal Fluminense, Niterói, Brazil.

^4^School of Pharmacy and Biomolecular Sciences, Liverpool John Moores University, Liverpool, United Kingdom.

*** Correspondence:**

Jose M. Prieto [j.m.prietogarcia@ljmu.ac.uk](mailto:j.m.prietogarcia@ljmu.ac.uk)

# Supplementary Figures

## HPLC-UV of the phytochemical standards


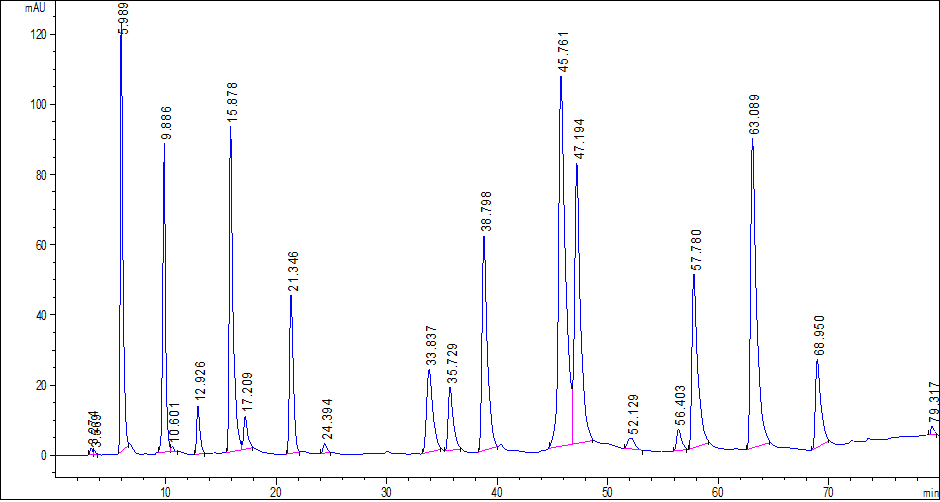


Gallic acid

2,4-dihydroxybenzoic acid

Caffeic acid

Epicatechin

Epigallocatechin

Catechin

Vitexin rhamnoside

Vitexin

Rutin

Ellagic acid

Quercitrin

Hesperidin

Quercetin

Luteolin

Kaempferol

**Supplementary Figure 1.** HPLC-UV chromatogram of the phytochemical standards (254 nm). Each standard was present in the mixed solution at a final concentration of 1mg/ml.

## HPLC-UV fingerprint of the preparation from medicinal plants **of Latin American tradition**

| **(a)**  254nm | ****** |
| --- | --- |
| **(b)** 280nm | ****** |
| **(c)**  360nm. | ****** |
| **Supplementary Figure 2.** HPLC-UV fingerprint of *Apuleia ferrea (Mart.) Baill (Fabaceae) Fruits Accepted name: Libidibia ferrea (Mart.) L. P. Queiroz*  (50 mg/mL) | |

| **(a)**  254nm | ****** |
| --- | --- |
| **(b)** 280nm | ****** |
| **(c)**  360nm. | ****** |
| **Supplementary Figure 3.** HPLC-UV fingerprint of *Bidens pilosa L.* (Asteraceae)  Aerial parts (50 mg/mL) | |

| **(a)**  254nm | ******  (**1)(2**) Phenolic acids |
| --- | --- |
| **(b)** 280nm | ****** |
| **(c)**  360nm. | ****** |
| **Supplementary Figure 4.** HPLC-UV fingerprint of *Casearia sylvestris* Sw. (Salicaceae)  Leaves (50 mg/mL) | |

| **(a)**  254nm | ****** |
| --- | --- |
| **(b)** 280nm | ****** |
| **(c)**  360nm. | ****** |
| **Supplementary Figure 5.** HPLC-UV fingerprint of *Costus spicatus* (Jacq.) Sw. (Costaceae) Leaves (50 mg/mL). | |

| **(a)**  254nm | ****** |
| --- | --- |
| **(b)** 280nm | ****** |
| **(c)**  360nm. | ****** |
| **Supplementary Figure 6.** HPLC-UV fingerprint of *Lippia sidoides* Cham. (Verbenaceae) Leaves (50 mg/mL) | |

| **(a)**  254nm | ****** |
| --- | --- |
| **(b)** 280nm | ****** |
| **(c)**  360nm. | ****** |
| **Supplementary Figure 7.** HPLC-UV fingerprint of *Maytenus ilicifolia* Mart. Ex Reissek (Celastraceae) Leaves. Accepted name *Monteverdia ilicifolia* (Mart. ex Reissek Biral) (50 mg/mL). | |

| **(a)**  254nm | ****** |
| --- | --- |
| **(b)** 280nm | ****** |
| **(c)**  360nm. | ****** |
| **Supplementary Figure 8.** HPLC-UV fingerprint of *Persea americana* Mill. (Lauraceae)  Leaves (50 mg/mL) | |

| **(a)**  254nm | ****** |
| --- | --- |
| **(b)** 280nm | ****** |
| **(c)**  360nm. | ****** |
| **Supplementary Figure 9.** HPLC-UV fingerprint of *Schinus terebinthifolia* Raddi (Anacardiaceae) Bark (50 mg/mL) | |

| **(a)**  254nm | ****** |
| --- | --- |
| **(b)** 280nm | ****** |
| **(c)**  360nm. | ****** |
| **Supplementary Figure 10.** HPLC-UV fingerprint of *Solidago microglossa DC.* (Asteraceae) Aerial parts, accepted name: *Solidago chilensis* Meyen (50 mg/mL). | |

| **(a)**  254nm | ****** |
| --- | --- |
| **(b)** 280nm | ****** |
| **(c)**  360nm. | ****  **(3-5)** Glycosilated flavonoids |
| **Supplementary Figure 11.** HPLC-UV fingerprint of *Syzygium jambolanum (Lam.) DC.* Fruits and seeds, accepted name: *Syzygium cumini* (L.) Skeels (50 mg/mL). | |

| **(a)**  254nm | ****** |
| --- | --- |
| **(b)** 280nm | ****** |
| **(c)**  360nm. | **** |
|  | **Supplementary Figure 12.** HPLC-UV fingerprint of *Tabebuia avellanedae Lorentz ex Griseb.* (Bignoniaceae) Bark. accepted name: *Handroanthus impetiginosus* (Mart. Ex DC.) Mattos (50 mg/mL) |

| **(a)**  254nm | ****** |
| --- | --- |
| **(b)** 280nm | ****** |
| **(c)**  360nm. | ****** |
| **Supplementary Figure 13.** HPLC-UV fingerprint of *Vernonia polyanthes Less.* (Asteraceae) Leaves, accepted name: *Vernonanthura phosphorica* (Vell.) H.Rob. (50 mg/mL). | |

## HPLC-UV fingerprint of the preparation from medicinal plants **of European tradition**

| **(a)**  254nm | **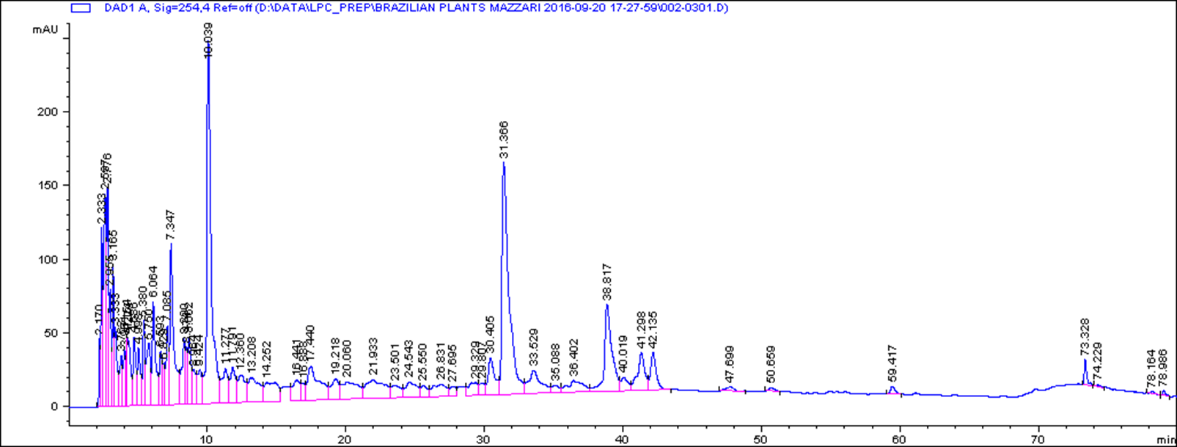** |
| --- | --- |
| **(b)** 280nm | ***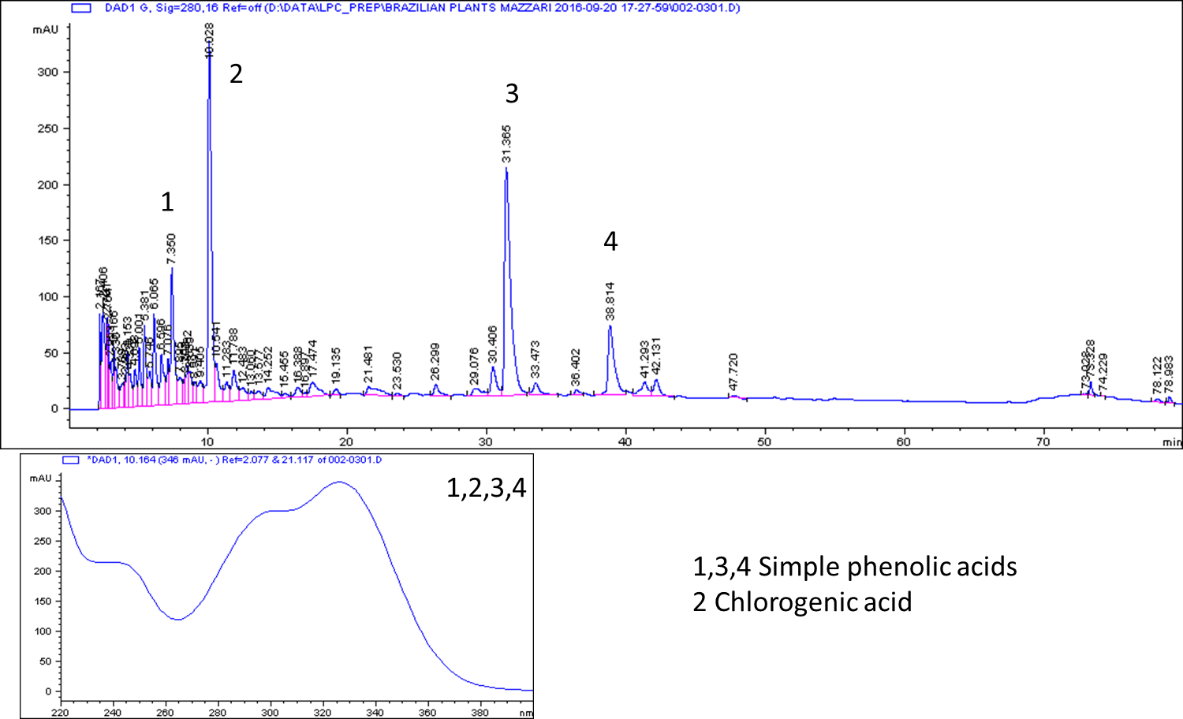*** |
| **(c)**  360nm. | ***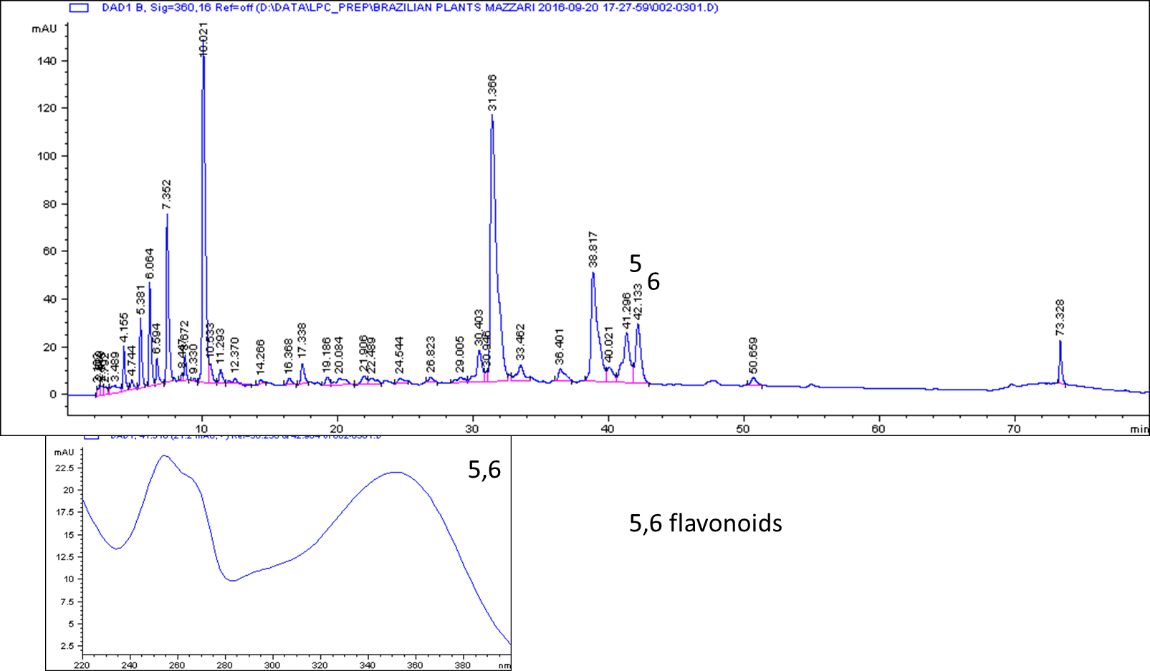*** |
| **Supplementary Figure 14.** HPLC-UV fingerprint of *Artemisia absinthium* L. (Asteraceae) Leaves or leafy flowering tops (50 mg/mL). | |

| **(a)**  254nm | **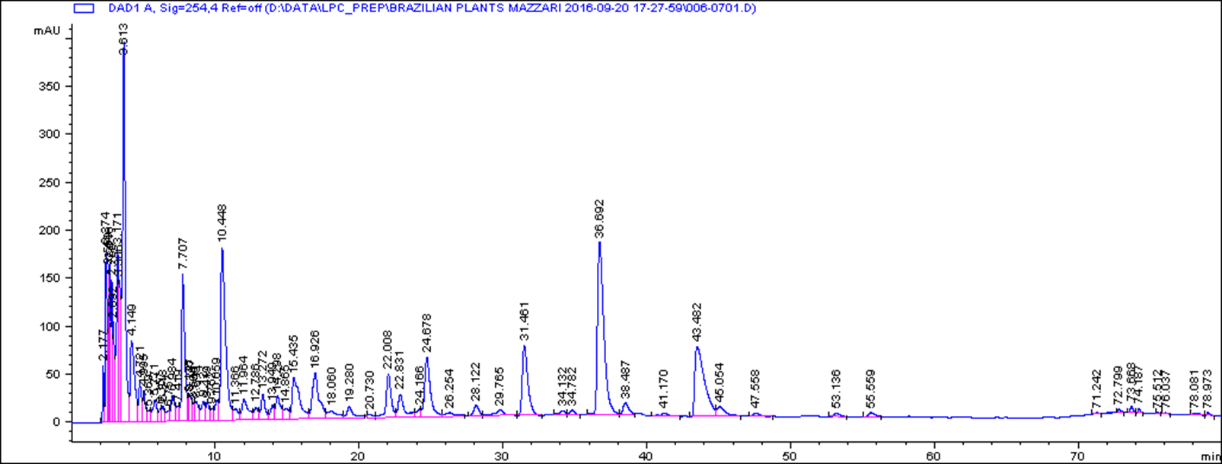** |
| --- | --- |
| **(b)** 280nm | ***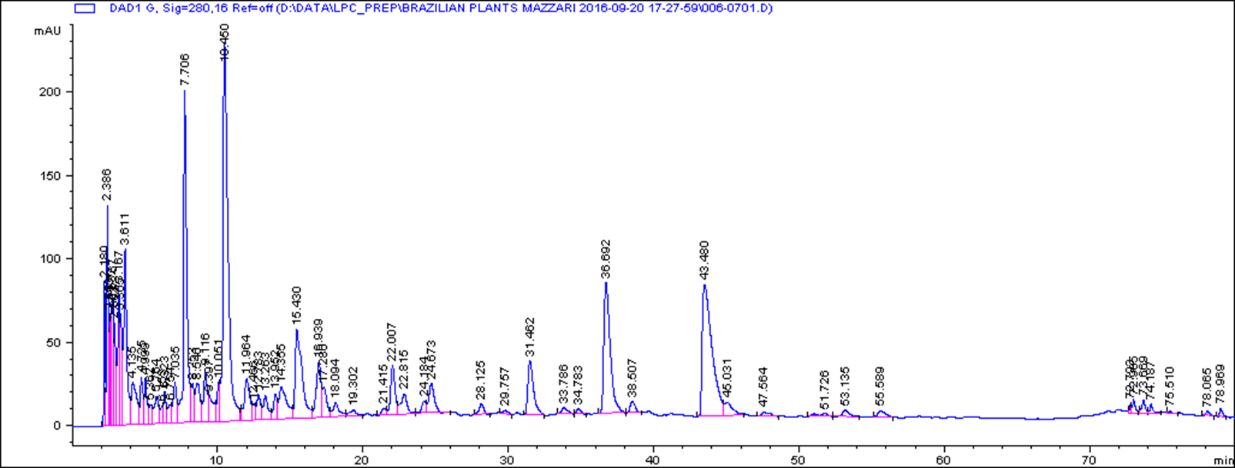*** |
| **(c)**  360nm. | ***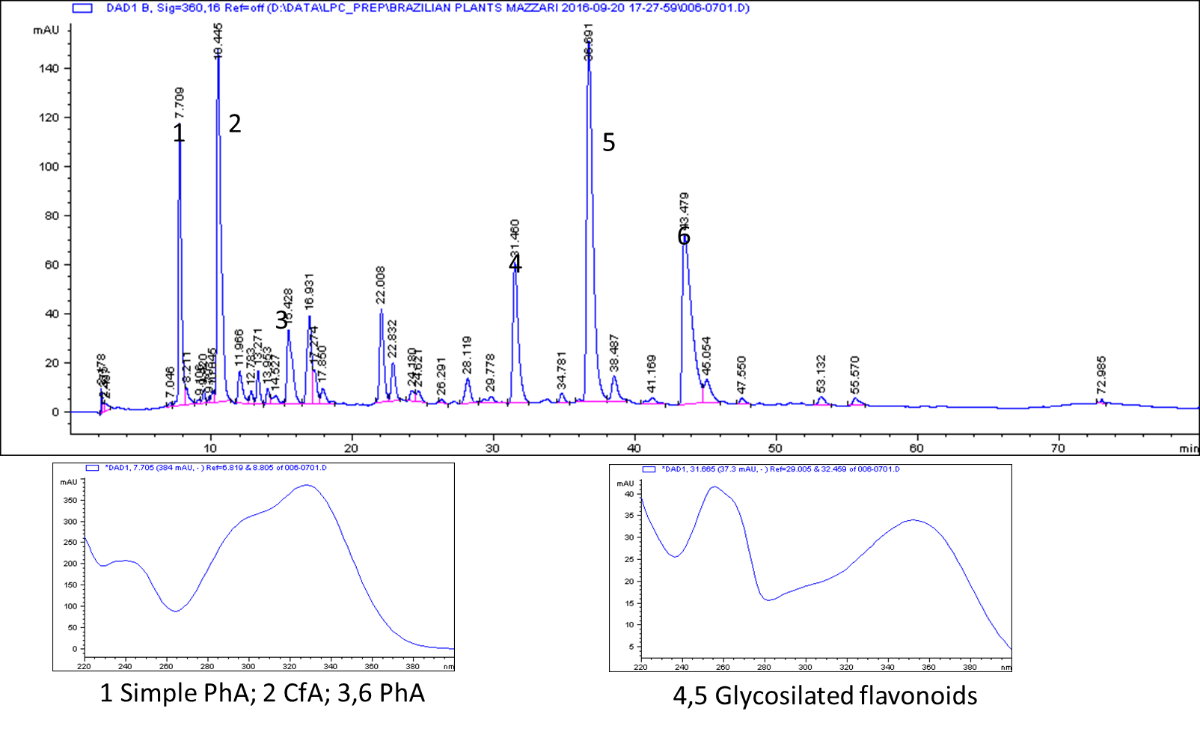*** |
| **Supplementary Figure 15.** HPLC-UV fingerprint of *Equisetum arvense* L. (Equisetaceae) Aerial parts (50 mg/mL). | |

| **(a)**  254nm | **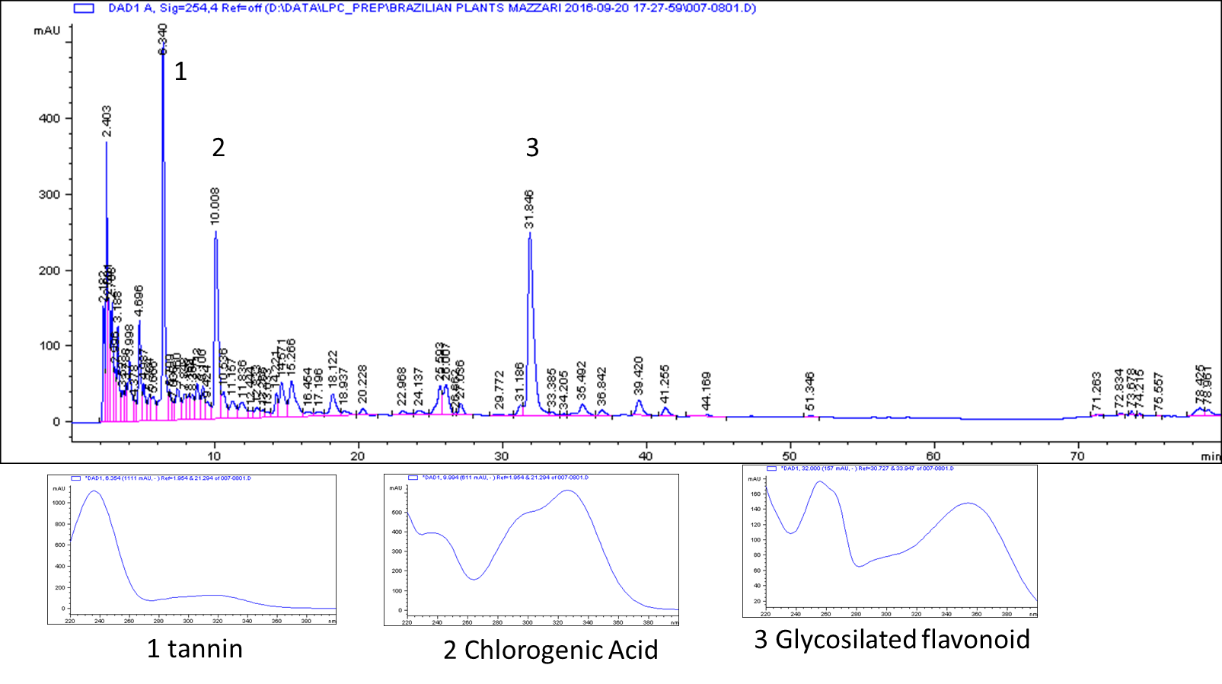** |
| --- | --- |
| **(b)** 280nm | ***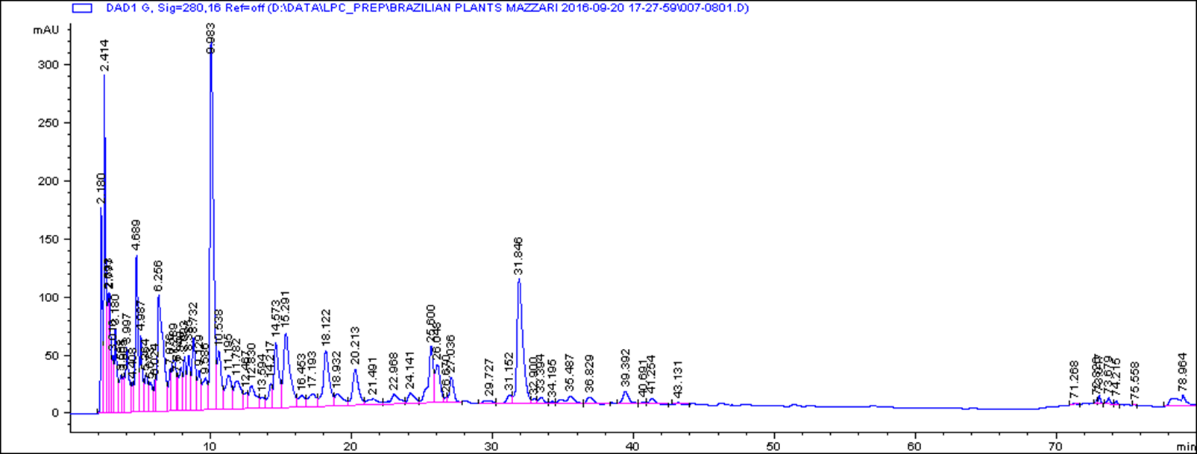*** |
| **(c)**  360nm. | ***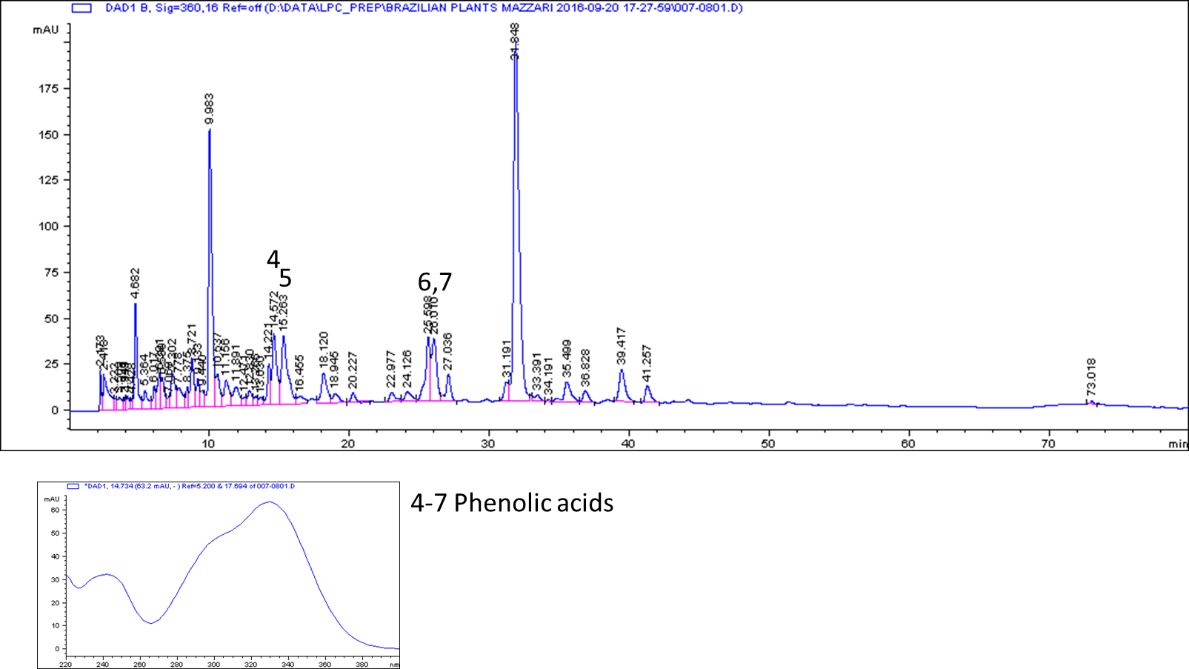*** |
| **Supplementary Figure 16.** HPLC-UV fingerprint of *Lamium album* L. (Lamiaceae) Leaves (50 mg/mL). | |

| **(a)**  254nm | ***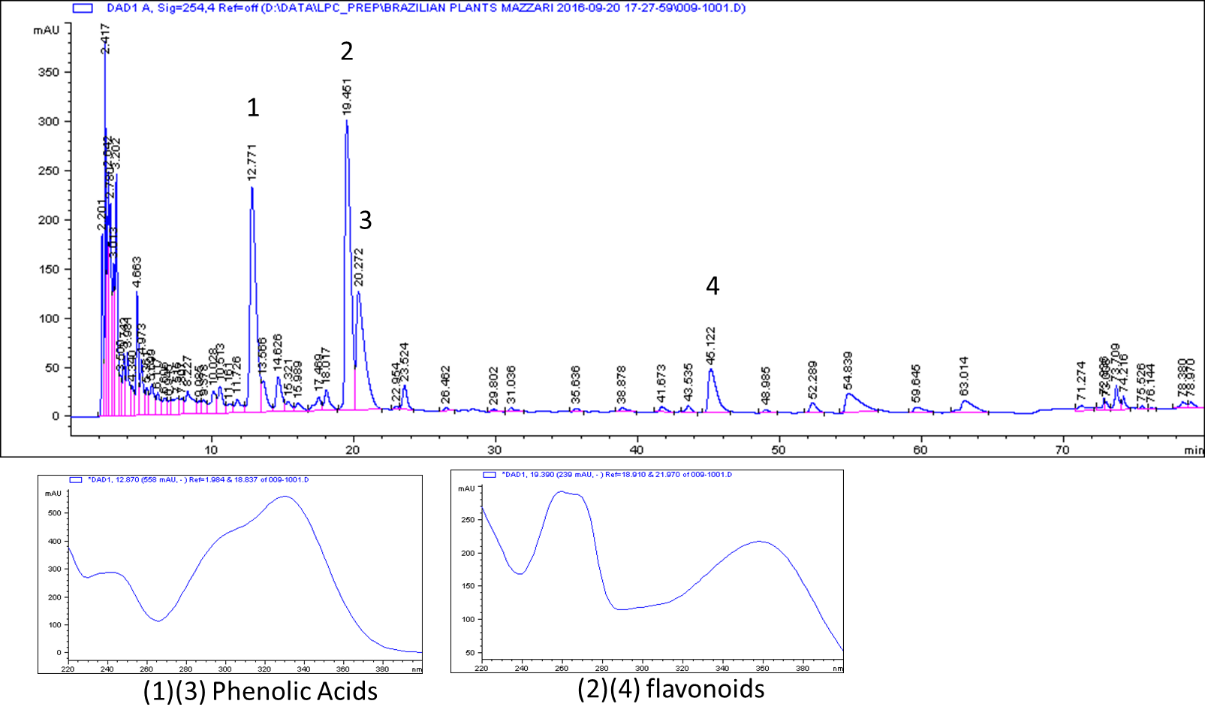*** |
| --- | --- |
| **(b)** 280nm | ***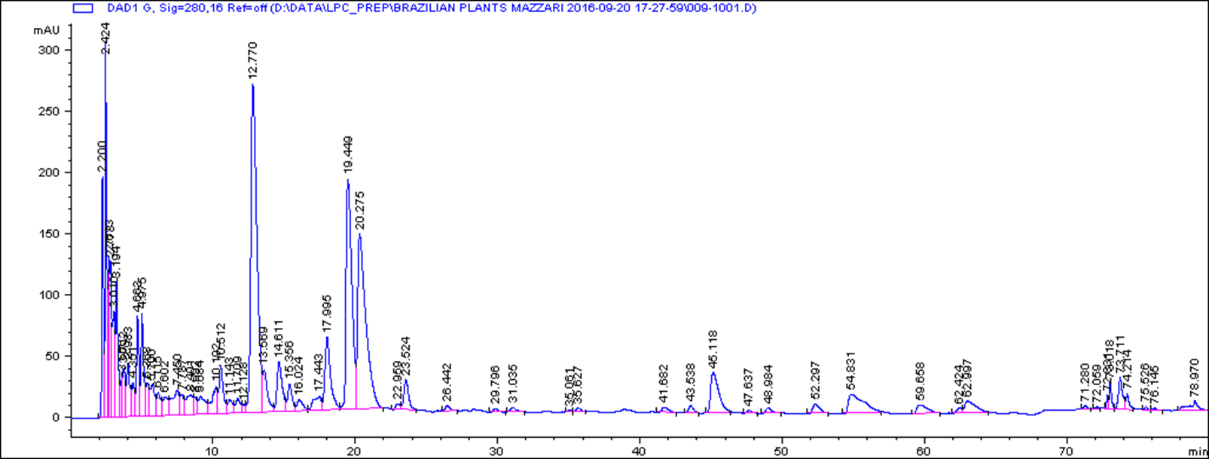*** |
| **(c)**  360nm. | ***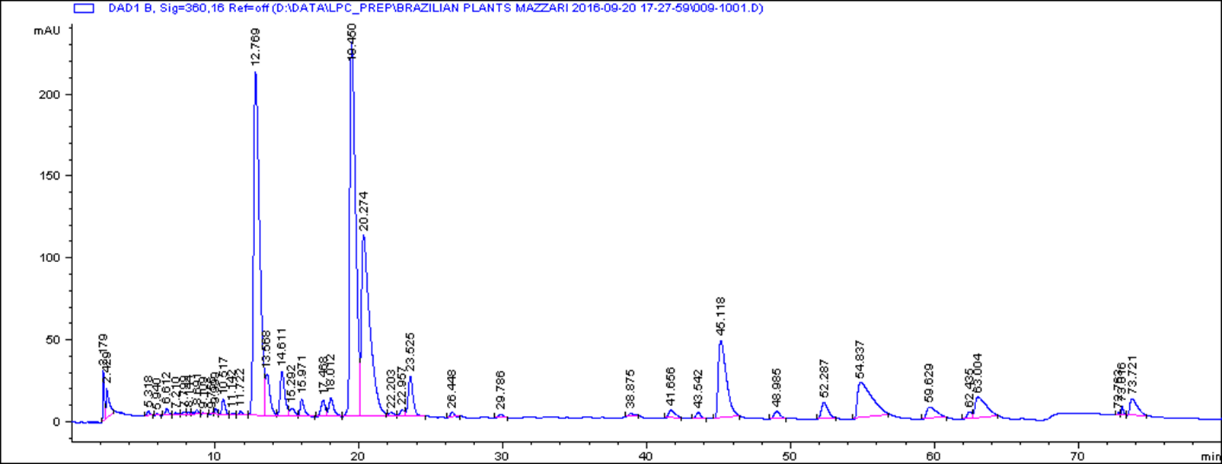*** |
| **Supplementary Figure 17.** HPLC-UV fingerprint of *Malva sylvestris* L. (Malvaceae) leaves (50 mg/mL). | |

| **(a)**  254nm | **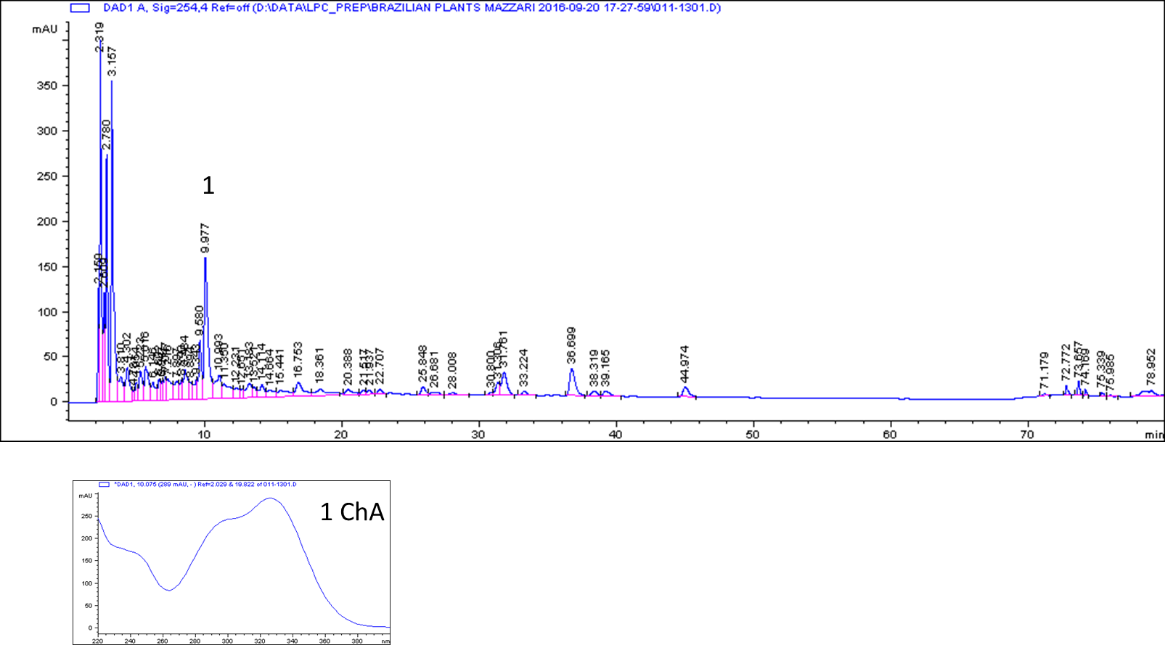** |
| --- | --- |
| **(b)** 280nm | ***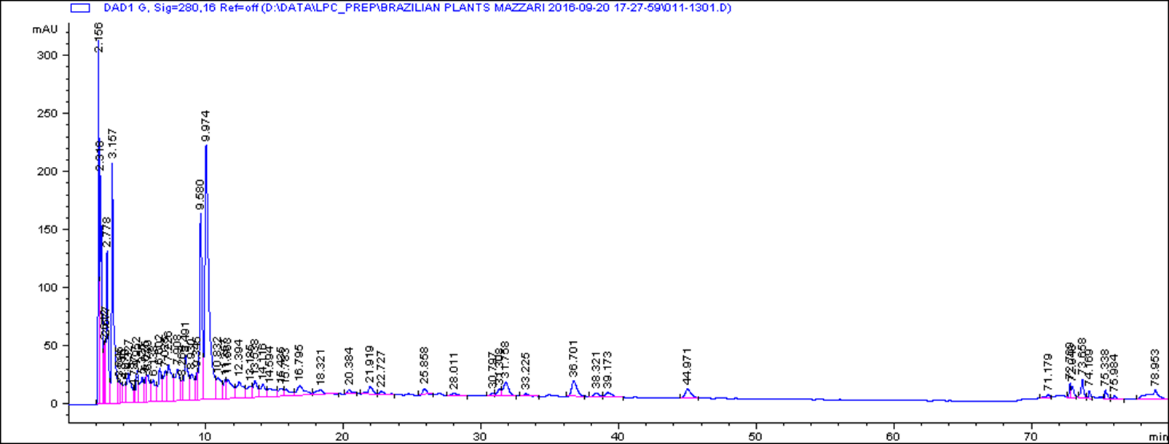*** |
| **(c)**  360nm. | ***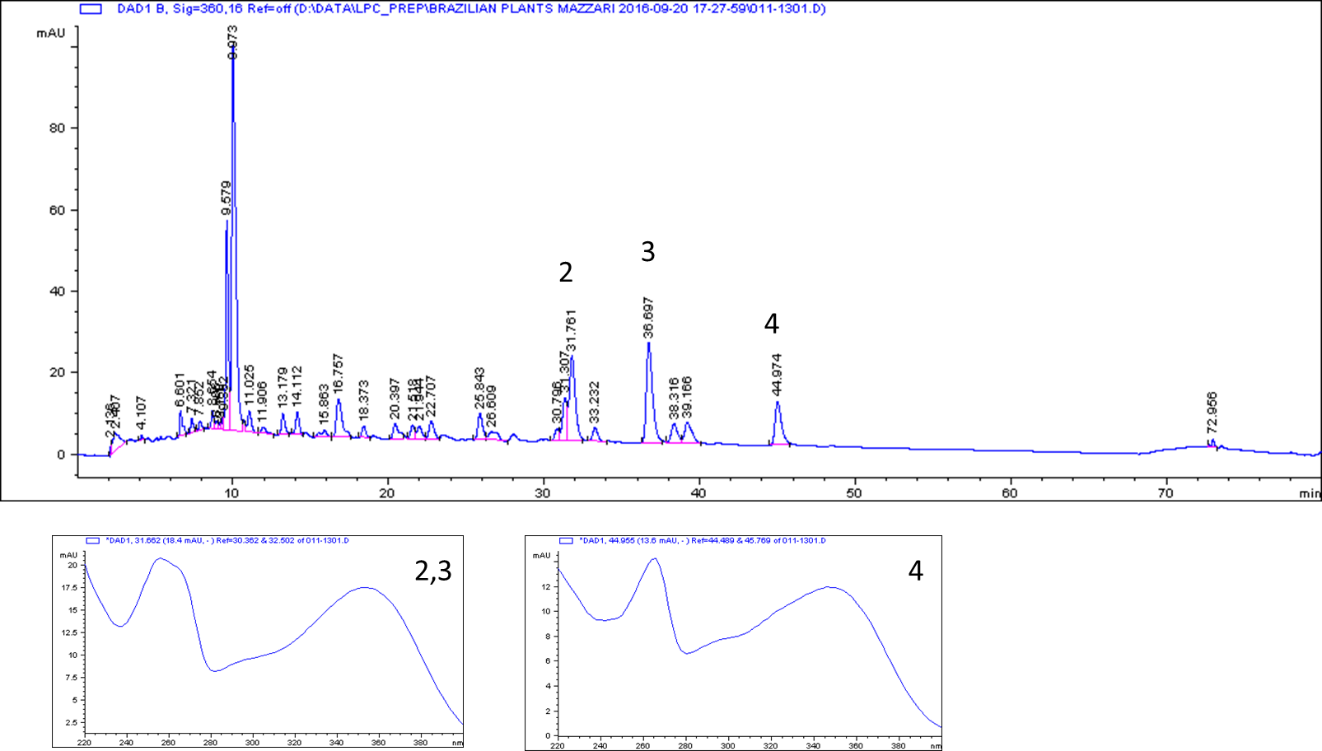*** |
| **Supplementary Figure 18.** HPLC-UV fingerprint of *Morus nigra* L. (Moraceae) Leaves (50 mg/mL). | |

| **(a)**  254nm | **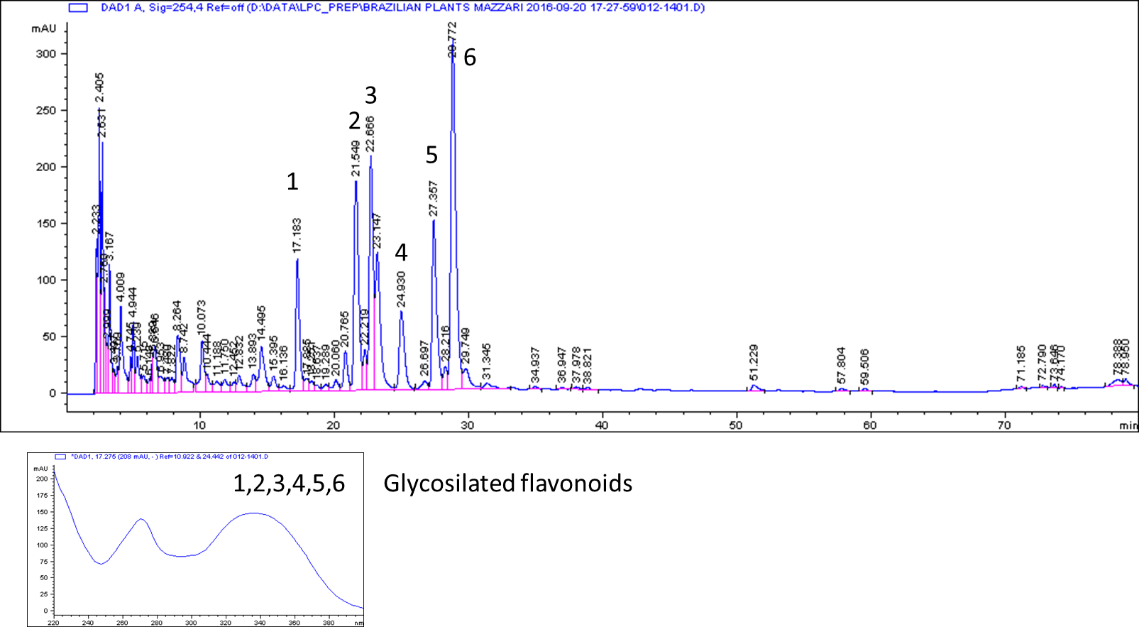** |
| --- | --- |
| **(b)** 280nm | ***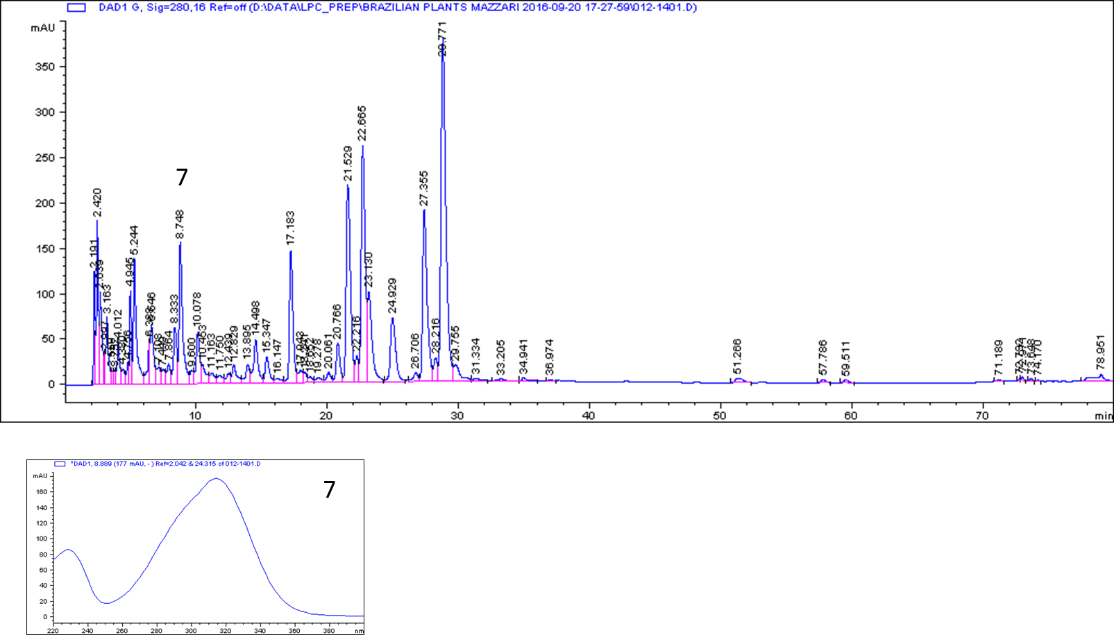*** |
| **(c)**  360nm. | ***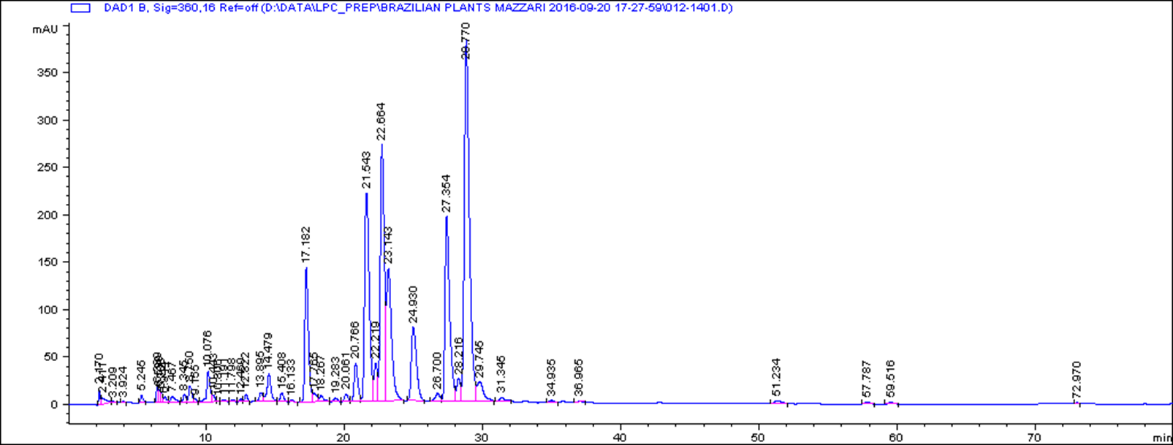*** |
| **Supplementary Figure 19.** HPLC-UV fingerprint of *Passiflora incarnata* L. (Passifloraceae) Aerial parts (50 mg/mL). | |

| **(a)**  254nm | **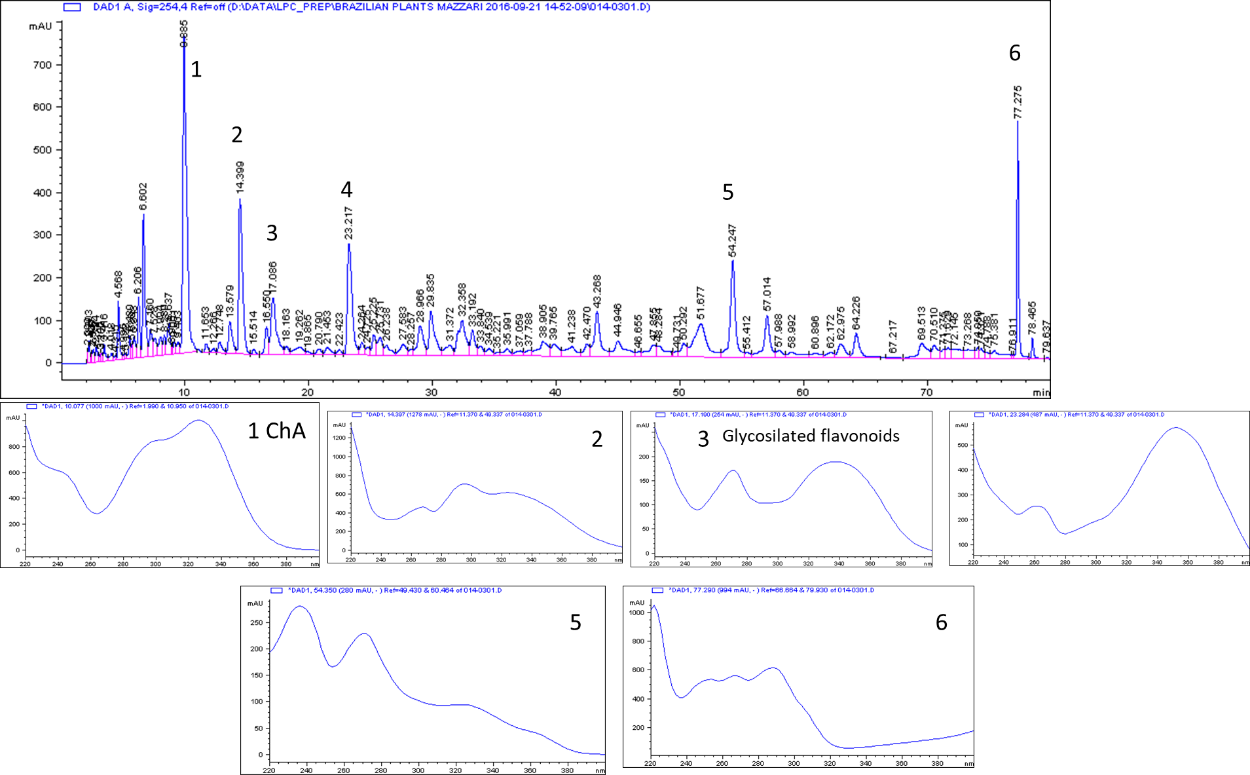** |
| --- | --- |
| **(b)** 280nm | ***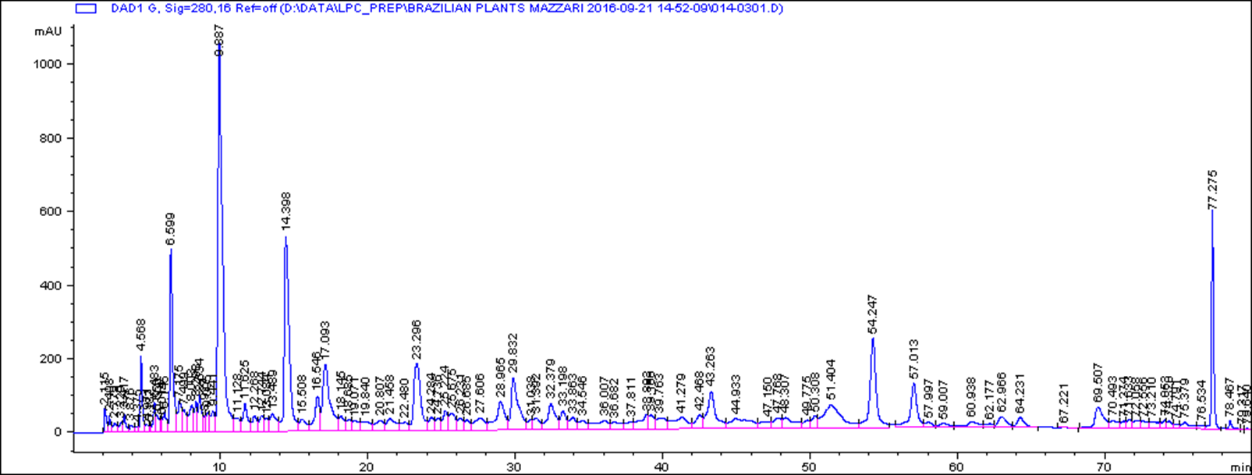*** |
| **(c)**  360nm. | ***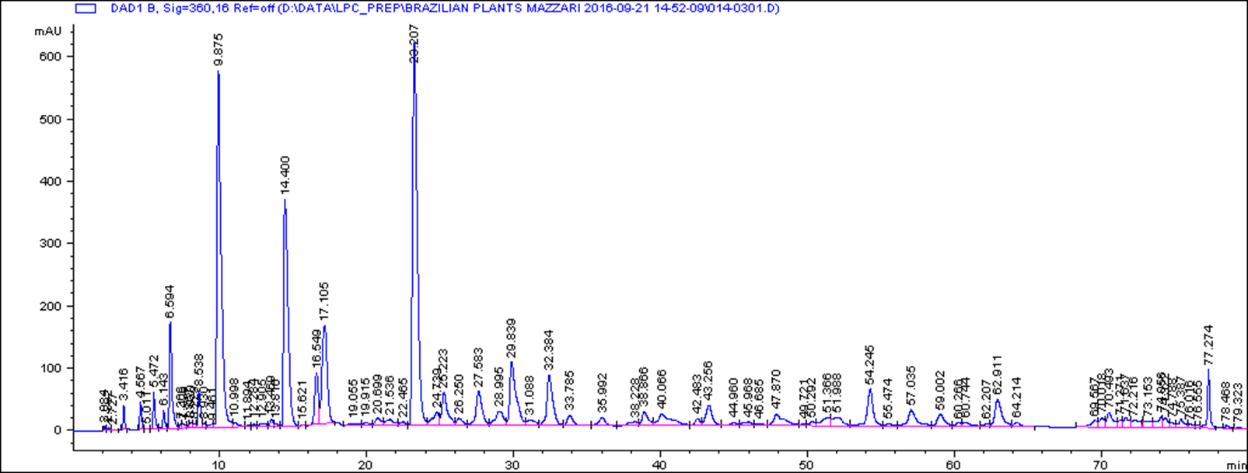*** |
| **Supplementary Figure 20.** HPLC-UV fingerprint of *Rhamnus purshiana L.* (Rhamnaceae) Accepted name: *Frangula purshiana* Cooper. Bark (50 mg/mL). | |

| **(a)**  254nm | **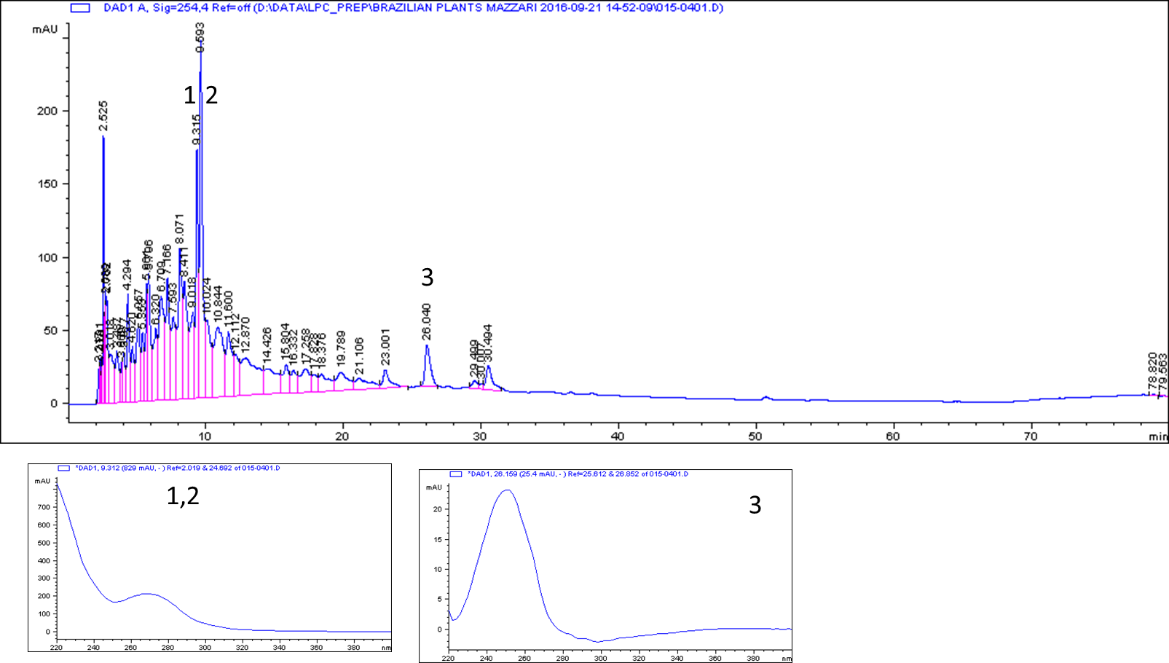** |
| --- | --- |
| **(b)** 280nm | ***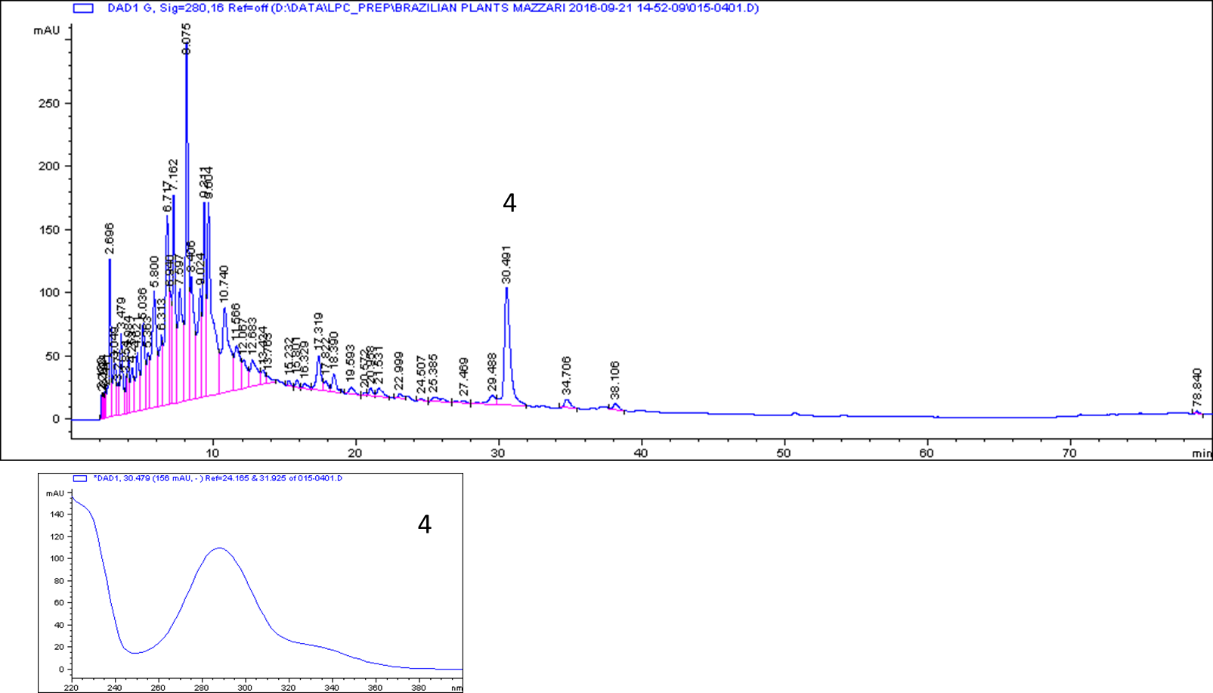*** |
| **(c)**  360nm. | ***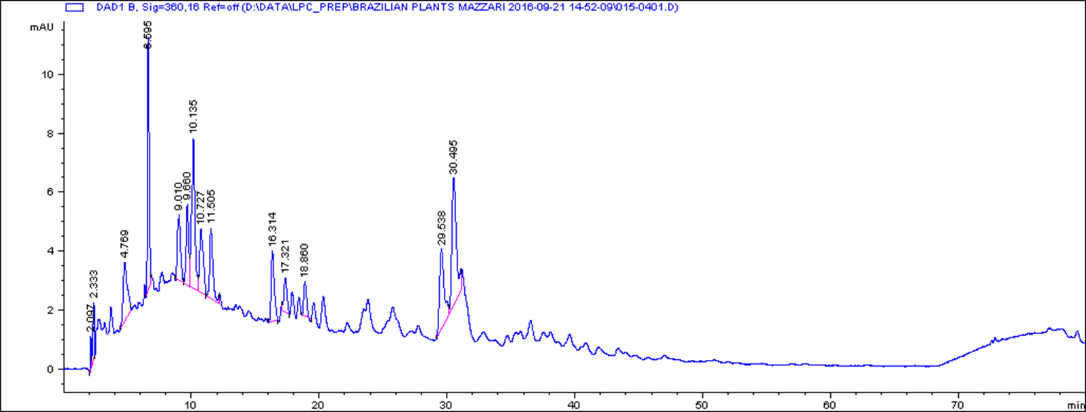*** |
| **Supplementary Figure 21.** HPLC-UV fingerprint of *Salix alba* L. bark (50 mg/mL). | |

**End of Supplementary materials**
